# CNPY4 inhibits the Hedgehog pathway by modulating membrane sterol lipids

**DOI:** 10.1038/s41467-022-30186-x

**Published:** 2022-05-03

**Authors:** Megan Lo, Amnon Sharir, Michael D. Paul, Hayarpi Torosyan, Christopher Agnew, Amy Li, Cynthia Neben, Pauline Marangoni, Libin Xu, David R. Raleigh, Natalia Jura, Ophir D. Klein

**Affiliations:** 1 Cardiovascular Research Institute, University of California San Francisco, San Francisco, CA 94158 USA; 2 Program in Craniofacial Biology and Department of Orofacial Sciences, University of California, San Francisco, CA USA; 3 The Institute of Biomedical and Oral Research, Faculty of Dental Medicine, Hebrew University, Ein Kerem, Jerusalem, Israel; 4 Department of Medicinal Chemistry, University of Washington, Seattle, WA USA; 5 Department of Radiation Oncology, University of California, San Francisco, San Francisco, CA USA; 6 Department of Neurological Surgery, University of California, San Francisco, San Francisco, CA USA; 7 Department of Cellular and Molecular Pharmacology, University of California San Francisco, San Francisco, CA 94158 USA; 8 Department of Pediatrics and Institute for Human Genetics, University of California, San Francisco, CA USA; 9 Department of Pediatrics, Cedars-Sinai Medical Center, Los Angeles, CA USA

**Keywords:** Sterols, Embryogenesis, Morphogen signalling, Membrane proteins

## Abstract

The Hedgehog (HH) pathway is critical for development and adult tissue homeostasis. Aberrant HH signaling can lead to congenital malformations and diseases including cancer. Although cholesterol and several oxysterol lipids have been shown to play crucial roles in HH activation, the molecular mechanisms governing their regulation remain unresolved. Here, we identify Canopy4 (CNPY4), a Saposin-like protein, as a regulator of the HH pathway that modulates levels of membrane sterol lipids. *Cnpy4*^*–/–*^ embryos exhibit multiple defects consistent with HH signaling perturbations, most notably changes in digit number. Knockdown of *Cnpy4* hyperactivates the HH pathway in vitro and elevates membrane levels of accessible sterol lipids, such as cholesterol, an endogenous ligand involved in HH activation. Our data demonstrate that CNPY4 is a negative regulator that fine-tunes HH signal transduction, revealing a previously undescribed facet of HH pathway regulation that operates through control of membrane composition.

## Abstract (web summary)

Although lipids are known to affect Hedgehog (Hh) signalling, the underlying mechanisms remain unclear. Here, the authors show that Canopy4 regulates membrane sterol lipid levels, with knockout mouse embryos exhibiting digit number changes and other Hh signalling-related developmental defects.

## Introduction

The hedgehog (*HH*) gene was first identified in *Drosophila* as a regulator of larval segmentation^1^, after which three mammalian homologs were discovered: desert hedgehog (*Dhh*), Indian hedgehog (*Ihh*), and sonic hedgehog (*Shh*)^2–5^. *Shh* is the most widely expressed HH ligand and is found in the epithelium and at epithelial–mesenchymal boundaries of various tissues, including the tooth, gut, lung, and limb, where it controls morphogenesis and adult homeostasis^6^. Precise regulation of *Shh* signaling is therefore critical for proper tissue development and patterning. Perturbations to the pathway have been linked to severe congenital abnormalities, including polydactyly and holoprosencephaly^7^, and numerous cancers^8^.

HH signal transduction in vertebrates occurs through a tightly regulated process at the primary cilium, an antenna-like organelle that protrudes from the surface of most cells^9,10^. Signaling is initiated by binding of a secreted HH ligand to the Patched 1 (PTCH1) receptor, which resides at the base of and within primary cilia^11–14^. HH binding to PTCH1 releases the inhibition of the G-protein-coupled receptor Smoothened (SMO), leading to SMO accumulation in cilia^15^. Activation of SMO releases the inhibition of the glioma-associated oncogene (GLI) transcription factors (GLI1, 2, and 3) by a negative regulator of the pathway, Suppressor of Fused (SUFU)^16^. This allows the GLI proteins to translocate into the nucleus and initiate transcription of key developmental genes^17–19^. HH activation also upregulates transcription of pathway genes including *Ptch1* and *Gli1*, leading to a complex signaling feedback loop^20,21^.

Several components of the HH pathway interact with sterol lipids, and both depletion of cellular lipids and inhibition of sterol biosynthesis hinder HH signal transduction^22–27^. These lipid molecules are thought to influence trafficking of proteins into and out of the cilia, thereby facilitating signaling from this organelle^10^. Furthermore, lipids can either directly modify components of the pathway, as is the case with HH ligands^28–32^, or allosterically activate them^33–36^. An example of the latter is regulation of SMO, which is thought to be endogenously activated by one or more sterol lipid ligands^34^. Thus, sterol localization and concentration at the plasma membrane is critically tied to HH signaling. Genetic perturbations of cholesterol biosynthesis can cause congenital disorders that prominently feature limb malformations^37^. Dysregulation of sterol homeostasis can additionally manifest as HH-associated cancers, such as medulloblastoma, the most common malignant brain cancer in children^8^.

Pharmaceutics that treat HH-driven cancers primarily target key nodes in the HH pathway, as is the case with the SMO inhibitors vismodegib and sonidegib. However, use of these therapeutics can be complicated by challenges, such as variable responsiveness, adverse side effects, and drug resistance^38–40^. The essential role of lipids in the regulation of HH signaling makes direct targeting of sterols or the enzymes responsible for their biosynthesis an attractive therapeutic alternative. Recent studies have suggested that statins, which reduce levels of cholesterol, may be a therapeutically viable option for HH-driven malignancies^41,42^. Specifically, simvastatin was shown to synergize with vismodegib to slow proliferation of cultured medulloblastoma cells^41,42^ and reduce adverse side effects on bone growth^42^. Although the tunability and specificity of statin treatment for HH-driven diseases remain to be determined, this promising therapeutic avenue underscores the need for the characterization of molecules that modulate HH signaling through sterols, as they could serve as potential drug targets.

Saposin and saposin-like (SAPLIP) proteins represent attractive candidates for regulators of signaling pathways modulated by lipids. SAPLIPs most commonly function via direct interaction with lipids and/or membranes to regulate (i) membrane binding, (ii) membrane lipid extraction, and (iii) membrane permeabilization^43^. The five Canopy proteins (CNPY1–5) comprise an understudied SAPLIP family of ER-resident proteins^44^, which have been linked to a variety of cellular processes related to intracellular signaling pathways. Specifically, *cnpy1* was found to positively regulate the expression of fibroblast growth factor (*fgf*) *8* in the midbrain–hindbrain region of zebrafish^45^ and the development of Kupffer’s vesicle^46^, which controls left–right asymmetry through HH signaling^47,48^. Like zebrafish cnpy1, CNPY2 has also been linked to FGF signaling, as its expression was positively correlated with FGF21 stimulation in human and mouse cells, and it was found to be important for FGF21-dependent expression of the low-density lipoprotein receptor (LDLR)^49^, to which PTCH1 is thought to be closely related^50^. In addition, CNPY2, perhaps the best studied member of the CNPY family, has been shown to possess a broad range of functions^51–56^, including regulation of neurite outgrowth in mouse neuroblastoma cells^51^, enhancement of angiogenesis^52^, and initiation of the PERK-CHOP pathway in human and mouse models^53^. The closely homologous CNPY3 and CNPY4, also referred to as proteins associated with Toll-like receptor 4 (PRAT4s), have been reported to up- or down-regulate the membrane levels of human TLRs, respectively^57–63^. Lastly, the recently identified CNPY5 protein may play a role in the folding of secretary proteins in the ER^44^. Given the diverse roles that CNPY proteins play in cellular signaling, there has been a growing interest in characterizing their biological function in animal models, with *Cnpy2* and *Cnpy3* knockout mice already reported^53,64^.

Here, we demonstrate the developmental consequences of *Cnpy4* knockout in mice and report the unique role that CNPY4 plays in the regulation of HH signaling. Deletion of *Cnpy4* in mice manifests in developmental defects consistent with those reported in mice with impaired HH signaling, including alterations to digit number. Knockdown of *Cnpy4* in cells results in hyperactivation of the HH pathway, as measured by a luciferase reporter assay for *Gli* transcription as well as *Gli1* mRNA transcript levels. This hyperactivation of the HH transcriptional pathway is dependent on SMO and is likely a result of elevated levels of accessible cholesterol throughout the membrane of cells lacking *Cnpy4*.

## Results

### Deletion of *Cnpy4* in mice leads to developmental defects consistent with perturbations to the HH pathway

**Fig. 1:**
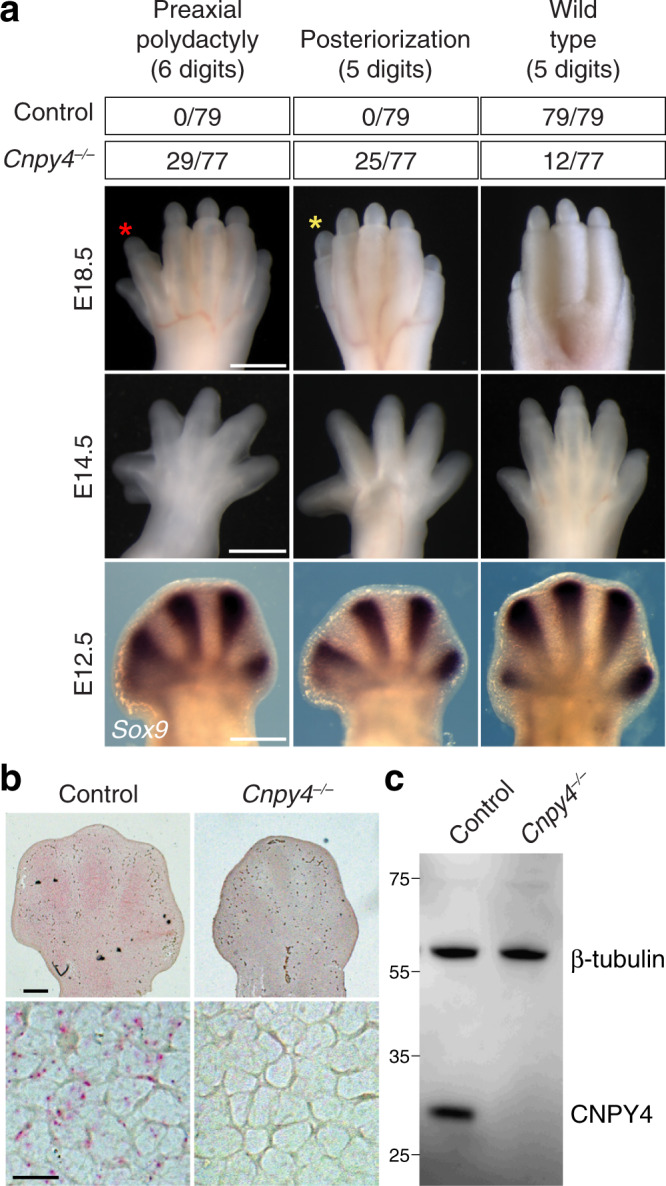
Developmental defects in *Cnpy4*^*–/–*^ hindlimbs. **a** Dorsal view of control and *Cnpy4* mutant hindlimbs at embryonic day (E)18.5 (top row) and E14.5 (middle row). The majority of *Cnpy4* mutant hindlimbs exhibit either an extra digit anteriorly (red asterisk) or a transformation of digit 1 from biphalangeal to triphalangeal (yellow asterisk). Whole mount in situ hybridization for *Sox9* at E12.5 (bottom row) indicates an extra digit and an enlarged digit 1 primordium. The top table summarizes the phenotype frequency in mutant hindlimbs; less frequent phenotypes are shown in Supplementary Fig. 1a, b. The scale bars represent 500 μm. **b** Single molecule in situ hybridization (RNAscope) of *Cnpy4* in the hindlimbs of control and *Cnpy4* mutant embryos at E12.5. The scale bars represent 200 μm (top) and 40 μm (bottom). **c** Protein levels in lysates of MEF cells from control and mutant embryos were detected using the indicated antibodies by Western blot analysis. All experiments were performed three independent times with similar results.

We first assessed the developmental role of CNPY4 by breeding *Cnpy4* null mice and analyzing the effect of CNPY4 loss of function in mutant embryos. We found a striking phenotype in the limbs of *Cnpy4* mutants: of the *Cnpy4*^*–/–*^ embryos examined, 85% exhibited abnormalities in hindlimb digit number, ranging from the formation of one or two supernumerary digits on the anterior side of the limb (termed preaxial polydactyly) to a loss of up to three posterior digits (Fig. 1a and Supplementary Fig. 1a, b). Similar bi-directional phenotypic changes to the limb buds have been observed in patients with mutations in HH pathway genes^65,66^. We confirmed that *Cnpy4* transcripts are present in the limb buds of wild-type mouse embryos at embryonic day (E)12.5 and that expression is detectable in the long bones at later stages (Fig. 1b and Supplementary Fig. 2). Both *Cnpy4* transcript and CNPY4 protein were absent from mutant embryos (Fig. 1b, c). *Cnpy4* has additionally been reported to be widely expressed in mice, with the highest levels detected in the lung, thymus, uterus, and spleen^57^. Approximately 20% of the *Cnpy4*^*–/–*^ embryos exhibited anomalies in several parts of the embryo consistent with HH pathway dysregulation^67,68^, including rostral and/or caudal neural tube closure defects, splayed vertebrae, and abnormal rib morphology with fusions and bifurcations (Supplementary Fig. 1c). Due to the high penetrance of the limb phenotype and the central role of *Shh* in controlling digit number, we focused our analysis on the limb abnormalities in *Cnpy4* null mice.

### Loss of *Cnpy4* hyperactivates the HH transcriptional pathway both in vivo and in cells

**Fig. 2:**
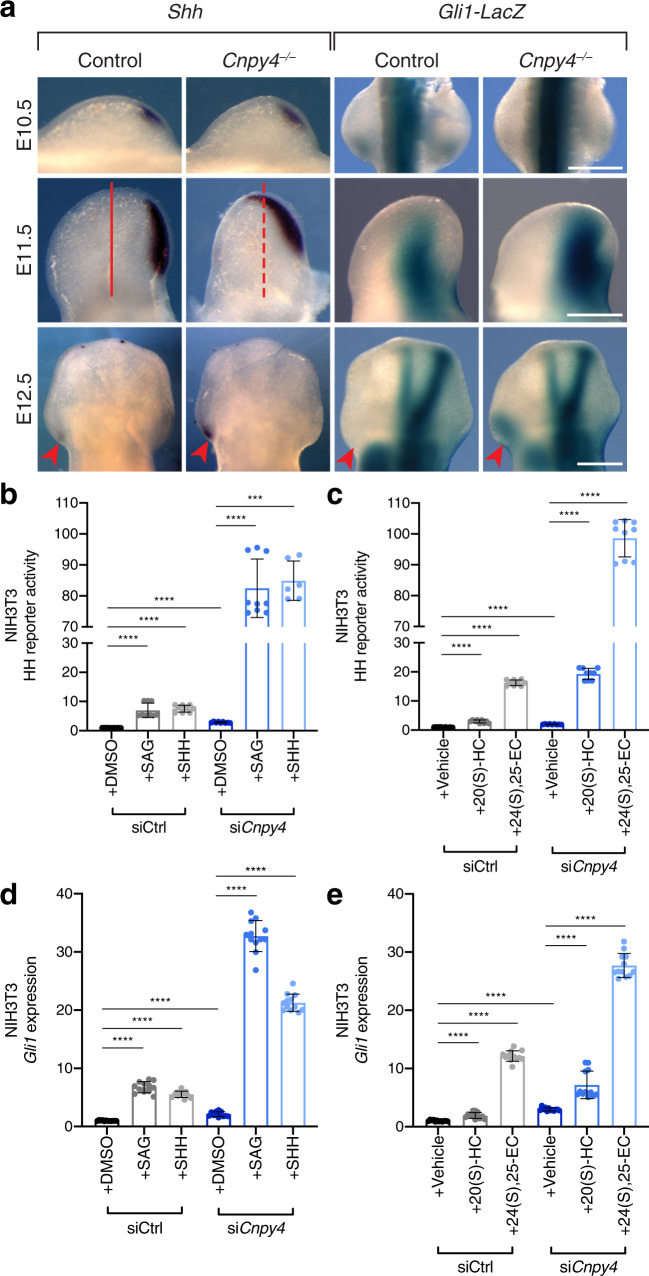
Absence of *Cnpy4* leads to hyperactivation of HH-related gene expression and signaling. **a** *Shh* in situ hybridization and *lacZ* expression of *Cnpy4;Gli1*^*lacZ*^ in hindlimb buds at embryonic day (E)10.5, E11.5, and E12.5. Samples at E11.5 show an enlarged *Shh* domain (lines) and samples at E12.5 have ectopic expression of both *Shh* and *Gli1* (arrowheads) in *Cnpy4* mutants. The scale bars represent 500 μm. **b**, **c** Luciferase reporter assay in ciliated NIH3T3 cells treated with control (gray bars) or *Cnpy4* (blue bars) siRNA and stimulated with SMO agonist (SAG), recombinant SHH (**b**), 20(S)-hydroxycholesterol (20(S)-HC), or 24(S), 25-exposycholesterol (24(S), 25-EC) (**c**). Data were normalized to the average value of control siRNA-treated cells stimulated with DMSO or vehicle. Data represent the mean ± SD (*n* = 9 from three biological and three technical replicates). Significance was calculated using a two-sided Mann–Whitney non-parametric test with ***p < 0.001 (*p*_si*Cnpy4*+SHH_ = 0.0004), ****p < 0.0001. **d**, **e** qRT-PCR assessment of *Gli1* expression in ciliated NIH3T3 cells treated with control (gray bars) or *Cnpy4* (blue bars) siRNA and stimulated with SAG, recombinant SHH (**d**), 20(S)-HC or 24(S), 25-EC (**e**). Data represent the mean ± SD (*n* = 12 from three biological and four technical replicates). Significance was calculated using a two-sided Mann–Whitney non-parametric test with ****p < 0.0001. All experiments were performed a minimum of three independent times with similar results.

To understand how *Cnpy4* modulates *Shh*, we examined the expression of *Shh* and its downstream effector *Gli1* during limb development in mutant embryos. *Shh* and *Gli1* expression expanded anteriorly in the early hindlimb buds of *Cnpy4* mutants (E11.5), and ectopic expression of *Shh* and *Gli1* was present in anterior domains at later developmental stages (E12.5) (Fig. 2a), in line with misactivation of the HH pathway. These changes are consistent with those observed in human patients and mouse models with preaxial polydactyly^69–71^. In a small number of mutants, reduction of *Shh* and *Gli1* expression was observed (Supplementary Fig. 3), paralleling the minority of *Cnpy4*^*–/–*^ mutants manifesting oligodactyly.

In order to quantify the *Cnpy4*-dependent changes in HH signaling at the cellular level, we utilized a luciferase reporter assay to measure *Gli* expression in NIH3T3 cells following transient *Cnpy4* knockdown with siRNA (Supplementary Fig. 4a). Consistent with the predominant polydactyly phenotype and other developmental abnormalities we observed in *Cnpy4* knockout embryos, silencing of *Cnpy4* resulted in elevated basal activation of the HH transcriptional program and potentiated signaling in response to HH pathway agonists (Fig. 2b, c). These effects were independent of the ligand used to activate the pathway and were observed upon stimulation with either a chemical SMO agonist (SAG), recombinant SHH, or synthetic (20(S)-hydroxycholesterol [20(S)-HC]) or cilia-associated (24(S), 25-epoxycholesterol [24(S), 25-EC]) oxysterols that bind to and activate SMO^35^. We corroborated these results by directly analyzing *Gli1* transcript levels in NIH3T3 cells using qRT-PCR. In line with the results from the HH luciferase reporter assay, we found that *Gli1* expression was greatly increased in *Cnpy4* knockdown cells compared to those treated with control siRNA upon ligand stimulation (Fig. 2d, e and Supplementary Fig. 4b, c). Thus, the HH pathway becomes hyperactivated in cells lacking *Cnpy4*, suggesting that CNPY4 is a negative regulator of the HH pathway.

### *Cnpy4* knockdown has little effect on ciliation and SMO localization

**Fig. 3:**
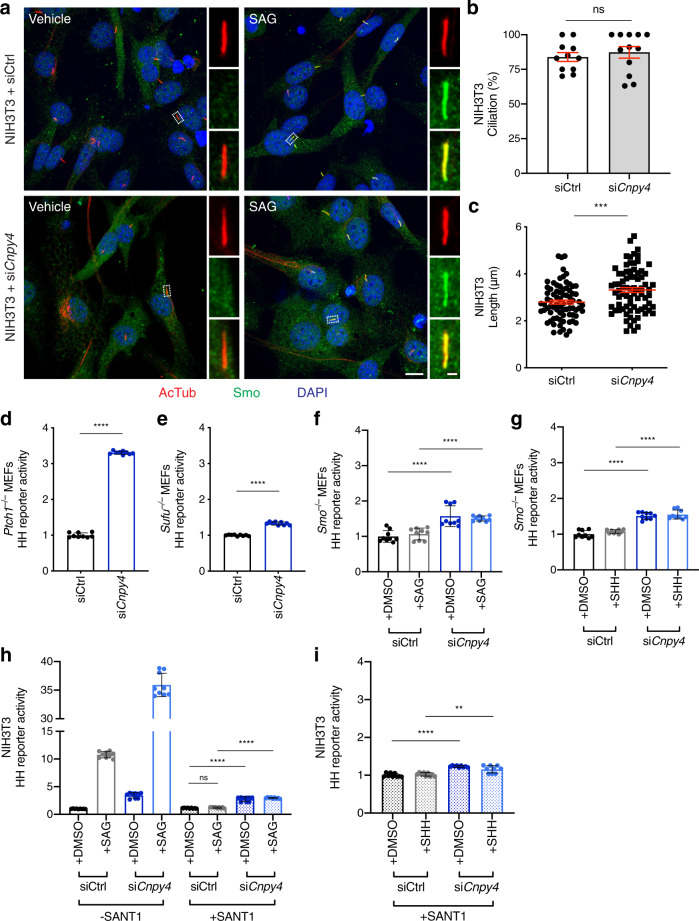
CNPY4 intersects the HH pathway at the level of SMO. **a** Immunofluorescence-based staining of primary cilia (acetylated tubulin, red), SMO (SMO, green), and nuclei (DAPI, blue) in ciliated NIH3T3 cells treated with control or *Cnpy4* siRNA. The scale bar represents 10 μm. Cilia scale bar represents 1 μm. **b** Quantification of the percentage of ciliated NIH3T3 cells, as assessed by acetylated tubulin immunofluorescence. Data represent the mean ± SEM (*n* = 152 siCtrl cells and *n* = 110 si*Cnpy4* cells from three biological replicates). Significance was calculated using a two-sided unpaired Welch’s *t*-test with ns *p* > 0.05 (*p* = 0.5286). **c** Quantification of the length of cilia in NIH3T3 cells. Measurements were made in FIJI using the acetylated tubulin channel. Data represent the mean ± SEM (*n* = 77 cells from three biological replicates). Significance was calculated using a two-sided unpaired Welch’s *t*-test with ***p < 0.001 (*p* = 0.0002). **d**–**g** Luciferase reporter assay in ciliated *Ptch1* (**d**), *Sufu* (**e**), or *Smo* (**f**, **g**) null MEFs treated with control (gray bars) or *Cnpy4* (blue bars) siRNA. *Smo* null MEFs were stimulated with either SAG (**f**) or SHH (**g**). **h**, **i** Luciferase reporter assay in ciliated NIH3T3 cells treated with control (gray bars) or *Cnpy4* (blue bars) siRNA stimulated with SAG (**h**) or SHH (**i**) in the presence of SMO antagonist (SANT-1). Data for **d**–**i** were normalized to the average value of control siRNA treated cells stimulated with DMSO where applicable. Data represent the mean ± SD (*n* = 9 from three biological and three technical replicates). Significance was calculated using a two-sided Mann–Whitney non-parametric test with ns *p* > 0.05 (*p*_siCtrl+SAG+SANT1_ = 0.0503), **p < 0.005 (*p*_si*Cnpy4*+SHH+SANT1_ = 0.0040), ****p < 0.0001. Experiments were performed a minimum of three independent times with similar results.

Morphological differences in cilia, changes in cell ciliation, and improper trafficking of ciliary proteins are all linked to aberrant HH activity during development^9,10,72,73^. We therefore asked if ciliary defects could explain the hyperactivation of the HH pathway. Cilia were assessed by staining for acetylated tubulin, a marker of the ciliary axoneme, in *Cnpy4*-deficient NIH3T3 cells and in mouse embryonic fibroblast (MEF) cells derived from *Cnpy4*^*–/–*^ embryonic limb buds (Fig. 3a and Supplementary Fig. 5a–c). *Cnpy4* knockdown NIH3T3 cells did not show significant differences in the percentage of ciliated cells compared to control cells (Fig. 3b), and *Cnpy4*^*–/–*^ MEF cells differed from controls by <10% (Supplementary Fig. 5d). Furthermore, the length and overall morphology of cilia were not visibly altered by depletion of *Cnpy4* (Fig. 3a, c and Supplementary Fig. 5c, e). The intensity of SMO staining in the cilia upon SAG stimulation was also largely unchanged in *Cnpy4* silenced cells, indicating that the ability of SMO to traffic into the cilia was not impaired (Supplementary Fig. 5f, g). Similar uncoupling of ciliary morphology and SMO trafficking from HH activation was recently reported upon ablation of the cholesterol biosynthesis enzyme DHCR7^74^. We therefore concluded that the effect CNPY4 exerts on the HH pathway was likely through signaling-specific events, rather than ciliary or protein compartmentalization abnormalities.

### Absence of *Cnpy4* leads to modest effects on FGF signaling

FGF and HH signaling cooperate to drive limb bud development^75,76^, and the FGF pathway is subject to modulation by other members of the CNPY family^45,46,49^. Thus, we asked whether FGF signaling is also altered in the absence of CNPY4 by measuring the levels of FGF-dependent phosphorylation of Akt and ERK in control and *Cnpy4*^*−/−*^ MEF cells (Supplementary Fig. 6). The maximum magnitude of FGF-induced Akt and ERK phosphorylation in *Cnpy4*^*−/−*^ MEFs was not statistically significantly different than that measured in the control MEFs, although the duration of Akt pathway activation was diminished in *Cnpy4*^*−/−*^ MEFs. Compared to the observed CNPY4-dependent modulation of HH signaling, these effects on FGF signaling duration appeared quite modest. While we cannot exclude a possibility that FGF signaling also contributes to the phenotypes in *Cnpy4* knockout animals, we concluded that these effects are predominantly a result of altered HH signaling.

### Hyperactivation of the HH pathway by knockdown of *Cnpy4* requires SMO

Next, to map the impact that CNPY4 exerts on HH signal transduction components, we utilized our in vitro system to perform epistasis experiments in *Ptch1*, *Smo*, or *Sufu* null MEFs. Although knockout of *Ptch1* alone constitutively activates the HH pathway^15^, knockdown of *Cnpy4* further activated the HH transcriptional program in *Ptch1*^–/–^ MEFs compared to control cells (Fig. 3d and Supplementary Fig. 7a–c), suggesting that CNPY4 intersects the HH pathway parallel to or downstream of PTCH1. Knockout of *Sufu*, a negative regulator of the pathway downstream of PTCH1, also results in constitutive activation of the HH pathway^77^. However, in contrast to the effect of *Cnpy4* knockdown in *Ptch1*^–/–^ MEFs, knockdown of *Cnpy4* in *Sufu*^–/–^ MEFs led to a comparatively modest increase of *Gli1* mRNA transcription (Fig. 3e and Supplementary Fig. 7d–f), suggesting that CNPY4 functions upstream of SUFU to inhibit HH signal transduction.

SMO functions in the HH pathway downstream from PTCH1 and upstream from SUFU^78^. As *Smo*^*–/–*^ MEFs are unable to transduce HH signals in response to pathway ligands, we examined whether the observed CNPY4-mediated modulation of HH signaling required SMO using both genetic (Fig. 3f, g and Supplementary Fig. 8a, b) and pharmacological (Fig. 3h, i and Supplementary Fig. 8c–e) perturbations. In the absence of SMO, SAG or recombinant SHH stimulation was unable to elicit hyperactive HH signaling even after *Cnpy4* knockdown (Fig. 3f, g and Supplementary Fig. 8b), indicating that, like PTCH1, CNPY4 modulation of HH activity requires SMO. This lack of hyperactivation was also observed in *Cnpy4*-silenced NIH3T3 cells when SMO was pharmacologically inhibited by its antagonist SANT-1, which directly competes with SAG for binding to SMO (Fig. 3h, i and Supplementary Fig. 8d). We note that these cells displayed slightly elevated levels of basal HH activity upon knockdown of *Cnpy4*, despite the absence or repression of SMO in these cells (Fig. 3f–i and Supplementary Fig. 8b, d), though to a much lesser extent than cells expressing SMO. Together, these findings point to an essential role of SMO in the ligand-dependent potentiating effect of CNPY4 loss on HH signaling.

### Recombinant CNPY4 does not bind ligands known to activate HH signaling

As SMO and PTCH1 are both transmembrane proteins whose signaling is likely sensitive to the local lipid environment^78^, we asked if CNPY4, as a SAPLIP protein, influences HH signaling by modulating the lipid composition of the membrane. In comparison to other membrane compartments, the ciliary membrane in which PTCH1 and SMO reside is highly enriched in cholesterol and oxysterols^35^. These lipids have been shown to directly bind and activate SMO^33–36^. We therefore probed the ability of CNPY4 to directly interact with cholesterol and several of these HH-activating oxysterol compounds in vitro. We purified a recombinant construct of human CNPY4 (hCNPY4ΔCt) lacking its signal sequence and C-terminal tail, which is predicted to be largely unstructured (Supplementary Fig. 9a). Purified hCNPY4ΔCt was well-folded and predominantly alpha helical, as expected for a SAPLIP protein^43,79^ (Supplementary Fig. 9b). However, under the conditions tested, recombinant hCNPY4ΔCt did not appear to bind cholesterol (Supplementary Fig. 9c, d). Furthermore, purified hCNPY4ΔCt did not display measurable binding to several oxysterols that are specifically enriched in the ciliary membrane and thought to be directly involved in HH pathway activation^35^ (Supplementary Fig. 9e). As the ability of many SAPLIP proteins to interact with lipids is directly tied to their dimerization^43,79^, we tested if CNPY4 is a dimer. Size-exclusion chromatography of recombinant hCNPY4ΔCt (Supplementary Fig. 9a) and co-immunoprecipitation between two differentially tagged constructs of full-length CNPY4 in cells (Supplementary Fig. 9e, f) are consistent with CNPY4 being a monomer. Thus, if CNPY4 does indeed modulate HH signaling by exerting control over membrane lipids, it likely does so via a novel mechanism.

### Elevation of membrane levels of accessible cholesterol likely underlies the ability of *Cnpy4* to hyperactivate the HH pathway

**Fig. 4:**
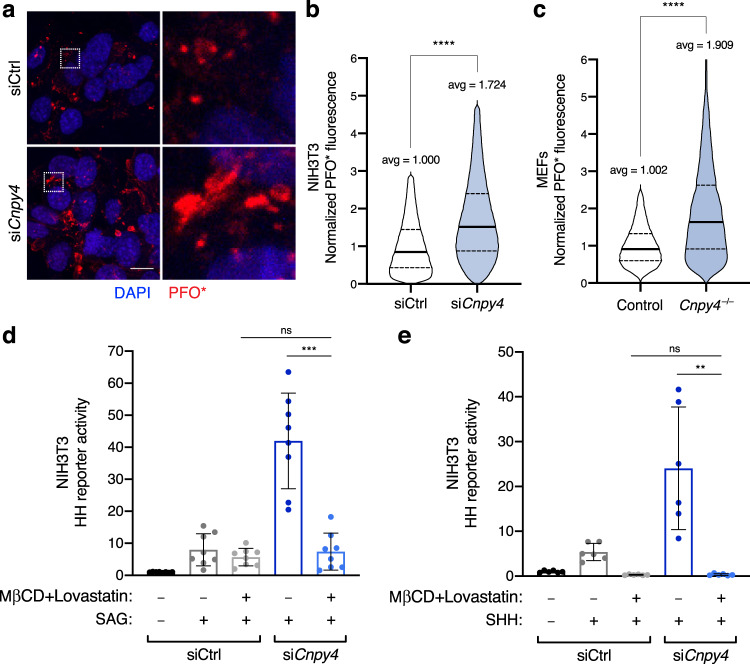
CNPY4 modulates levels of accessible cholesterol. **a** Immunofluorescence-based staining of accessible cholesterol (PFO*-AF647, red) and nuclei (DAPI, blue) in NIH3T3 cells treated with control or *Cnpy4* siRNA. Boxed areas are magnified on the right. The scale bar represents 10 μm. **b**, **c** FACS analysis of NIH3T3 cells treated with control or *Cnpy4* siRNA (**b**) or control and *Cnpy4*^*–/–*^ MEFs (**c**) stained with PFO*-AF647 for accessible cholesterol. Data were normalized to the average value of control siRNA treated or control cells. Data represent the distribution with the median (solid line) and the first and third quartiles (dashed lines) indicated (*n* = 244,550 siCtrl NIH3T3 cells, 291,375 si*Cnpy4* NIH3T3 cells, 92,183 control MEF cells, and 74,848 for *Cnpy4*^*–/–*^ MEF cells from two independent experiments with two biological replicates each). Significance was calculated using a two-sided unpaired Welch’s *t*-test with ****p < 0.0001. **d**, **e** Luciferase reporter assay in ciliated NIH3T3 cells, incubated with methyl-β-cyclodextrin (MCβD) and lovastatin prior to treatment with control (gray bars) or *Cnpy4* (blue bars) siRNA treatment and stimulation with SMO agonist (SAG) (**d**) or recombinant SHH (**e**). Data were normalized to the average value of control siRNA treated cells stimulated with vehicle. Data represent the mean ± SD (*n* = 9 from three biological and three technical replicates). Significance was calculated using a two-sided Mann–Whitney non-parametric test with ns *p* > 0.05 (*p*_siCtrl+SAGvs.si*Cnpy4*+SAG_ = 0.7984; *p*_siCtrl+SAGvs.si*Cnpy4*+SHH_ = 0.9069), **p < 0.005 (*p*_si*Cnpy4*+SHH_ = 0.0022), ***p < 0.001 (*p*_si*Cnpy4*+SAG_ = 0.0002). Experiments were performed three independent times with similar results.

Cholesterol is a major sterol lipid in eukaryotic plasma membranes, and it was recently proposed to be an endogenous ligand responsible for SMO activation^80^. We therefore evaluated whether CNPY4 modulates the membrane levels of unbound, accessible sterols, including cholesterol^81^. To directly measure the levels of accessible sterols in the plasma membrane of intact cells, we used a fluorescently tagged probe derived from the bacterial toxin Perfringolysin O (PFO*), which specifically recognizes these lipids^81^. Remarkably, NIH3T3 cells in which *Cnpy4* was knocked down displayed significantly elevated levels of accessible sterols compared to control treated cells (Fig. 4a, b and Supplementary Fig. 10). Likewise, MEFs derived from embryonic limb buds of *Cnpy4* null animals had notably increased levels of accessible sterols in a basal state (Fig. 4c and Supplementary Fig. 10). These data led us to hypothesize that the activating effect that CNPY4 loss exerts on the HH pathway is a consequence of increased levels of sterol lipids at the plasma membrane and thus would be eliminated if cholesterol was to be independently depleted from the membrane. To test this hypothesis, we pre-treated NIH3T3 cells with methyl-β-cyclodextrin (MβCD) prior to knockdown of *Cnpy4*. Cells were then maintained in a low concentration of MβCD and lovastatin to continuously inhibit cholesterol synthesis during *Cnpy4* knockdown, ciliation, and HH pathway stimulation with SHH or SAG. Strikingly, we found that in the absence of cholesterol, *Cnpy4* knockdown no longer potentiated HH signaling, irrespective of the stimulating ligand (Fig. 4d, e). We therefore concluded that the ability of *Cnpy4* knockdown to hyperactivate HH signaling is indeed due to an increase in membrane sterol levels.

**Fig. 5:**
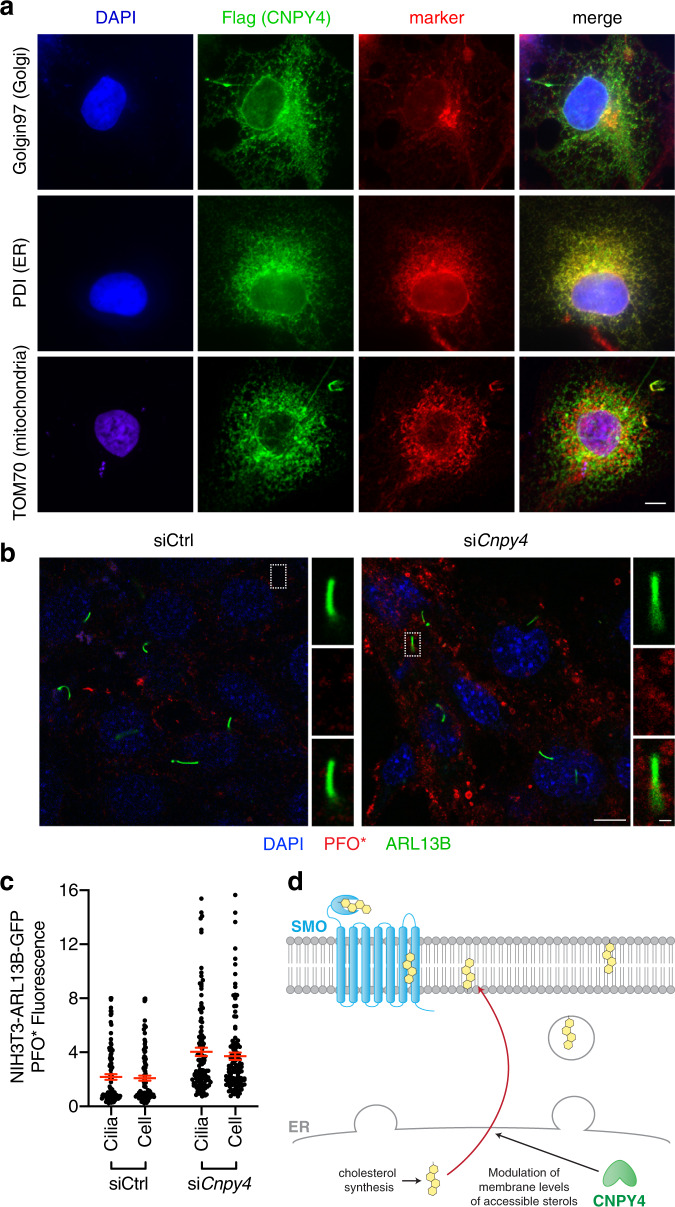
CNPY4 is an ER-resident protein that elevates membrane levels of accessible cholesterol. **a** Immunofluorescence-based staining of CNPY4 (Flag, green), organelle markers (indicated antibody, red), and nuclei (DAPI, blue) in COS-7 cells transiently transfected with Flag-tagged human CNPY4. The scale bar represents 10 μm. **b** Immunofluorescence-based staining of primary cilia (ARL13B-GFP, green), accessible cholesterol (PFO*-AF647, red), and nuclei (DAPI, blue) in ciliated NIH3T3 cells stably expressing ARL13B-GFP, treated with control or *Cnpy4* siRNA. The scale bar represents 10 μm. Cilia scale bar represents 1 μm. **c** Quantification of PFO* fluorescence intensity at the cilia and in the rest of the cell as described in the “Methods” section. Data represent the mean ± SEM (*n* = 104 siCtrl cells and *n* = 116 for si*Cnpy4* cells from three independent experiments). **d** Schematic illustrating proposed model of CNPY4 modulation of HH activation. CNPY4, an ER-resident protein, regulates the ability of unbound sterols synthesized in the ER to be expressed at cell membrane, thus controlling the activation of SMO.

Previous studies have reported that CNPY proteins localize to the ER^44^, and our immunostaining analysis of overexpressed Flag-tagged human CNPY4 in COS-7 cells corroborated these reports (Fig. 5a). We therefore asked whether CNPY4 alters the levels of accessible membrane lipid sterols via influencing sterol biosynthesis in the ER. We measured the effect *Cnpy4* knockdown had on global lipid sterol levels using a mass spectrometry-based sterolomics approach^82^ in NIH3T3 cells (Supplementary Fig. 11). Knockdown of *Cnpy4* led to decreases in the cellular levels of the cholesterol precursors 7-dehydrodesmosterol (7-DHD) and 7-dehydrocholesterol (7-DHC) and only modest increases in the level of cholesterol itself (Supplementary Fig. 11c, e, f). However, we observed no significant changes in the level of other cholesterol precursors, including desmosterol, which was also found to activate the HH pathway^83^ (Supplementary Fig. 11a, b, d). While it is possible that CNPY4 regulates the levels of specific cholesterol precursors, it did not appear to broadly regulate cholesterol biosynthesis. Thus, we reasoned that CNPY4 is likely rather involved in altering the distribution and/or accessibility of sterols to the components of the HH pathway. Using PFO* staining of NIH3T3 cells stably expressing GFP labeled ARL13B, a primary cilium marker, we examined if this redistribution is specific to the ciliary compartment in comparison to other cellular membranes. Knockdown of *Cnpy4* resulted in a marked increase of PFO* fluorescence in these cells (Fig. 5b), consistent with our previous findings in wild-type NIH3T3 cells. Interestingly, we observed the signal to be equally elevated in both the cilium and rest of the membrane of *Cnpy4* knockdown NIH3T3 cells (Fig. 5c), indicating that the increase in accessible sterols in *Cnpy4* knockdown cells does not occur specifically at the ciliary membrane.

## Discussion

Regulation of the HH pathway, which is among the most critically important signaling pathways in development^7,78^, remains poorly understood at the molecular level. Here, we have identified the SAPLIP CNPY4 as a negative regulator of the HH pathway. Germline deletion of CNPY4 in mice manifests as numerous developmental malformations consistent with perturbations to HH signaling^65–68^, most notably changes in digit number. In line with these observations, the expression of key components in the HH pathway, including the *Shh* morphogen and downstream effector molecule *Gli1*, are spatially misregulated at mid-embryonic stages in the absence of CNPY4. Intriguingly, we observed what appeared to be both up- and down-regulation of HH signaling in *Cnpy4*^*–/–*^ mice, as evidenced by embryos presenting both polydactyly and oligodactyly; this phenotypic variation will be an important future direction to investigate. We did not observe these bimodal data effects in our in vitro assays, in which CNPY4 consistently acted as a negative regulator of the HH pathway.

Our data indicate that *Cnpy4* knockdown likely modulates SMO-dependent HH activation by altering sterol lipid levels in the membrane, without directly affecting trafficking of SMO to the cilia. Increasing evidence supports the important role of sterol lipids in signal transduction between PTCH1 and SMO^22–27^; however, the molecular mechanisms governing these effects have yet to be fully elucidated. Recent studies have shown that both PTCH1 and SMO have several binding sites for sterols and that a subset of these binding events is essential for SMO activation^83–89^. PTCH1 was proposed to indirectly inhibit SMO by sequestering these activating sterols away^78,90^. This is thought to occur indirectly and to involve a proposed function of PTCH1 as a cholesterol pump, resulting in altered lipid composition of the plasma membrane^78,88^. Our data suggest that CNPY4 may also regulate composition of membrane sterols to fine-tune SMO-dependent HH activation (Fig. 5d), although likely through a mechanism distinct from that of PTCH1. Our data also reveal that, by doing so, deletion of *Cnpy4* can bypass the PTCH1-inhibition of HH-activation. Intriguingly, we additionally observed that depletion of *Cnpy4* causes a SMO-independent increase in basal HH activity, suggesting that accessible sterol levels in the membrane may contribute to HH signal transduction through multiple avenues. Recent work has illustrated that SMO movement into the cilia occurs even in the absence of ligand stimulation, albeit at much slower rates likely limited by diffusion^91,92^, and that sterol biosynthesis enzymes may regulate the ability of SMO to accumulate by priming the cilia via synthesis of sterols^74^. It is therefore possible that accessible sterol lipids are affected by *Cnpy4* knockdown and may play such a role.

How CNPY4 may regulate levels of accessible sterols at the plasma membrane remains an open question. CNPY4, as an ER-resident SAPLIP, is well positioned to assist with the synthesis, maturation, and membrane trafficking of lipids, such as sterols. The decreases in the levels of 7-DHC and 7-DHD and the slight increase in cholesterol, while possibly indicative of CNPY4’s ability to directly modulate lipid synthesis, could also indicate changes to the cholesterol biosynthesis enzyme DHCR7, which was recently shown to be positively correlated with HH activation^74^. CNPY4, rather than broadly regulating sterol biosynthesis, may instead influence the maturation and/or membrane trafficking of cholesterol synthesis and HH pathway components, such as DHCR7 or SMO, via control over sterol lipid distribution. Such a role for CNPY4 in receptor trafficking was previously reported for the regulation of TLR membrane expression^57,63^. While SAPLIP proteins have been shown to directly interact with lipids in the membrane^43,79^, in our hands CNPY4 does not bind to common sterol lipids. However, it is possible that full-length CNPY4 maintains this functionality, which we were unable to recapitulate using recombinant, truncated CNPY4. With the growing interest in treating HH-mediated malignancies through control of cholesterol levels, as evidenced by the repurposing of statins^41,42^, the seemingly specific effect of CNPY4 on the HH pathway could be of interest for design of a targeted therapeutic approach. Intriguingly, CNPY2 is reported to be necessary for the FGF21-dependent stimulation of LDLR expression^49^, which is also the target for statins^93^, further indicating that CNPY molecules may be a viable target for pharmaceutics to modulate HH signaling. In summary, our findings lay a foundation for unraveling the cellular mechanisms underlying CNPY4 signaling and for understanding the biological functions of other CNPYs and their potential roles as novel therapeutic targets.

## Methods

### Mouse breeding

Mice were maintained in the University of California, San Francisco (UCSF) specific pathogen-free animal facility in accordance with the guidelines established by the Institutional Animal Care and Use Committee (IACUC) and Laboratory Animal Resource Center (LARC). All experimental procedures were approved by the LARC at UCSF. Mice were maintained in 70 °F, 50% humidity temperature-controlled barrier facilities under a 12–12 h light cycle with access to food and water ad libitum. *Cnpy4* heterozygote mice were produced by Lexicon (http://www.lexicon-genetics.com). The resulting offspring were *129*-*C57BL*/*6J* hybrids and the line was backcrossed 12 generations to *C57BL*/*6J* (Jax: 000664) to make a congenic strain. *Gli1*^*LacZ*^ line (MGI: 2449767)^94^ was used to generate *Cnpy4*^*–/–*^*;Gli1*^*LacZ*^ embryos. To generate embryos at specific time points, adult mice were mated overnight, and females were checked for a vaginal plug in the morning. The presence of a vaginal plug was designated E0.5.

### Micro-computed tomography

Whole embryo or limb buds were collected and dehydrated through an ethanol series up to 70% ethanol. Samples were soaked in 1% phosphotungstic acid overnight to differentially stain soft tissues as previously described^95^ and scanned using a MicroXCT-200 (Carl Zeiss Microscopy) at 60 kV and 200 μΑ. We obtained 1200 projection images, taken at a total integration time of 3 s with linear magnification of ×2 and a pixel size of 6.4 μm. The volume was reconstructed using a back projection filtered algorithm (Zeiss). Following reconstruction, tissues were manually segmented and rendered as 3-D surfaces using Avizo Lite v9.1.1 (Thermo Fisher Scientific).

### Whole-mount in situ hybridization

Digoxygenin-labeled RNA probes (Roche) were generated by in vitro transcription from plasmids containing fragments of murine *Shh* (pBluescript II SK with a 640 bp Shh cDNA insert, courtesy of the McMahon lab) and *Sox9* (pBluescript II KS with a 250 bp *Sox9* cDNA insert, courtesy of the Lefebvre lab). Samples were fixed in 4% PFA overnight at 4 °C, and the hybridization was carried out as previously described^3^.

### RNAscope in situ hybridization

An RNAscope 2.5 HD Red (ACD, 322350) detection kit was used according to the manufacturer’s instructions. Sections were boiled in the target retrieval solution at ~100 °C for 15 min and incubated in the Protease Plus solution at 40 °C for 15 min. *Mus musculus Cnpy4* probe (475121 (lot # 16182A)) was used.

### Whole-mount *X-gal* staining

*LacZ* expression from *Cnpy4;Gli1*^*LacZ*^ embryos was detected by X-gal staining. Embryos were fixed for 45 min in 4% PFA at 4 °C, washed three times in rinse buffer containing 0.01% deoxycholate, 0.02% NP-40, 2 mM MgCl_2_, and 5 mM EGTA at room temperature and stained for 1 h at 37 °C in rinse buffer supplemented with 1 mg/mL X-gal, 5 mM K_3_Fe(CN)_6_, and 5 mM K_4_Fe(CN)_6_.

### Cell culture and drug treatments

Cells were cultured in Dulbecco’s modified Eagle medium (DMEM) (Thermo Fisher Scientific) supplemented with 10% fetal bovine serum (FBS) (Cytiva Life Sciences) and penicillin streptomycin (Thermo Fisher Scientific) and incubated at 37 °C with 5% CO_2_. All cell lines were tested quarterly for mycoplasma contamination using the MycoAlert mycoplasma detection kit (Lonza). Cells were stimulated with either 100 nM SAG (Merck Millipore), 1 μg/mL recombinant human SHH (C24II) N-terminus (R&D Systems), 30 μM 20(S)-hydroxycholesterol (Cayman Chemicals), 30 μM 24(S), 25-epoxycholesterol (Avanti Polar Lipids), or 25 μM SANT-1 (Selleck Chemicals). Incubations with SAG, SHH, and SANT-1 were done for 24 h, and oxysterols were done for 30–36 h. All stimulations were performed in low-serum OptiMEM (Thermo Fisher Scientific) to induce ciliation. For cholesterol depletion, cells were treated with 8 mM methyl-β−cyclodextrin (MβCD) (Sigma-Aldrich) for 45 min the night before siRNA knockdown. The media was then replaced with one supplemented with 1 mM MβCD and 5 μM lovastatin (Selleck Chemicals).

### Antibodies

The following primary antibodies were used in this study: beta-tubulin 1:2000 for WB (#2128, rabbit; Cell Signaling Technology); PRAT4B/CNPY4 1:500 for WB (AF5015, goat; R&D Systems); acetylated tubulin 1:1000 for IF (T6793, mouse, clone 6-11B-1; MilliporeSigma); Smoothened 1:1000 for IF (ab80683, rabbit; Abcam); Golgin97 1:100 for IF (A-21270, mouse, clone CDF4; Thermo Fischer Scientific); PDI 1:500 for IF (NB300-517, mouse, clone RL90; Novus Biologicals); TOM70 1:500 for IF (sc-17764, mouse, clone F-10; Santa Cruz Biotechnology); Akt-pS473 1:2000 for WB (#9271, rabbit; Cell Signaling Technology); Akt 1:2000 for WB (#9272, rabbit; Cell Signaling Technology); phospho-ERK1/2 1:2000 for WB (#9101, rabbit; Cell Signaling Technology); ERK1/2 1:2000 for WB(#9102, rabbit; Cell Signaling Technology); FLAG 1:2000 for WB (F1804, mouse, clone M2; MilliporeSigma); FLAG 1:500 for IF, 1:1000 for WB (#2368, rabbit; Cell Signaling Technology); and HA 1:500 for WB (sc-7392, mouse, clone F-7; Santa Cruz Biotechnology). The following fluorescently-labeled secondary antibodies were used in this study: Goat anti-Mouse IgG (H + L) Cross-Adsorbed Secondary Antibody, Alexa Fluor 647 1:500 for IF (A-21235; Thermo Fischer Scientific); Goat anti-Rabbit IgG (H + L) Highly Cross-Adsorbed Secondary Antibody, Alexa Fluor Plus 488 1:500 for IF (A32731; Thermo Fischer Scientific); and Goat anti-Mouse IgG (H + L) Cross-Adsorbed Secondary Antibody, Alexa Fluor 568 1:500 for IF (A-11004; Thermo Fischer Scientific). The following secondary antibodies were used in this study: Veriblot for IP detection reagent (HRP) 1:2000 for WB (ab131366; Abcam); Anti-rabbit IgG, HRP-linked Antibody 1:5000 for WB (#7074; Cell Signaling Technology); and Cytiva’s Amersham ECL Mouse IgG, HRP-linked whole Ab 1:5000 for WB (from sheep) (NA931; Cytiva Life Sciences). The HA, FLAG M2, FLAG antibodies were validated through empty vector transfection. The PRAT4B/CNPY4 antibody was validated through knockout and siRNA knockdown followed by Western blotting (loss of band of expected size). The beta-tubulin, acetylated tubulin, Smoothened, Golgin97, PDI, TOM70, Akt-PS473, Akt, phospho-ERK1/2, and ERK1/2 antibodies were validated through the manufacturer. Secondary antibodies were validated through no primary antibody controls.

### MEF generation

Embryos were isolated and washed in 1×PBS twice. Limb buds were separated using sterile tweezers from each embryo and washed with DMEM before incubation with 0.25% Trypsin/EDTA (Thermo Fisher Scientific) at 37 °C for 10 min. Trypsin was quenched by addition of DMEM supplemented with FBS and penicillin streptomycin. Cells were pipetted up and down at least 10 times to further dissociate cells before being transferred into fresh 15 mL tubes. Cells were gently pelleted at 200×*g* for 5 min at room temperature. Supernatant was carefully aspirated, and cells were resuspended in fresh media and plated in 6-cm plates. Additional cell debris was aspirated, and fresh media added daily until cells reached confluency, upon which they were split and expanded once before being pooled and flash frozen.

### siRNA transfection

22.5 pmol of siRNA SMARTpool (Horizon Discovery) was transiently transfected into indicated cells using lipofectamine RNAiMax (Thermo Fisher Scientific) according to the manufacturer’s protocol. Cells were transfected for 72 h before cell analysis. Confirmation of mRNA silencing was performed by qRT-PCR analysis, and confirmation of protein knockdown was analyzed via Western blotting.

### qRT-PCR analysis

NIH3T3 (ATCC) or MEF cells were grown in either 6- or 12-well plates and treated with the indicated expression conditions. RNA was extracted from cells using the RNEasy Mini kit (Qiagen) and reverse-transcribed to produce cDNA using the iScript cDNA synthesis kit (Bio-Rad) on a T100 thermal cycler (Bio-Rad) according to the manufacturers’ protocols. Target genes were amplified using PowerUp SYBR Green Master Mix (Thermo Fisher Scientific) on a QuantStudio6 thermocycler (Thermo Fisher Scientific). mRNA transcript relative abundances were calculated using the ΔΔCt method against *Gapdh*. Primers used for qRT-PCR analysis are listed in Supplementary Table 1 in  Supplementary Information.

### Luciferase-based reporter assays

NIH3T3 (ATCC) cells were plated in 6-well plates and transfected with siRNA as described above at least 16 h post-plating. 396 ng of Gli1-responsive Firefly luciferase reporter plasmid, 4 ng of a control Renilla luciferase reporter plasmid under the control of a constitutively active TK promoter, and 1 μg of pcDNA3.1+ empty vector were transfected into cells at least 6 h post-siRNA transfection using lipofectamine LTX with Plus reagent (Thermo Fischer Scientific) according to the manufacturer’s protocol. 16 h after DNA transfection, cells were recovered in fresh media for 24 h. Stimulation with the indicated ligand was performed in low-serum OptiMEM media (Thermo Fischer Scientific) for 24–36 h. Luciferase assays were conducted using the Dual Luciferase Reporter Assay System (Promega) and measured on a GloMax 96 Microplate Luminometer with Dual Injectors (Promega).

### Immunofluorescence-based staining

NIH3T3 (ATCC) or COS-7 (ATCC) cells were plated onto glass coverslips and transfected the following day using lipofectamine 3000 (Thermo Fischer Scientific) according to the manufacturer’s protocol. Cells were fixed in 3.7% PFA solution diluted in 1×PBS at room temperature with rocking for 15 min and then incubated with a 0.1% Triton X-100 and 2.5% BSA solution in 1×PBS to permeabilize cells and to block for non-specific antibody interactions for 30 min. Primary antibodies were diluted in blocking buffer and incubated overnight at 4 °C then washed out three times with 0.1% Triton X-100 in 1×PBS. Secondary antibodies were diluted in blocking buffer and incubated for 2 h at room temperatures before subsequent washes. DAPI staining was conducted for 10 min following the last wash before cells were mounted onto glass coverslips with Prolong Gold AntiFade Mountant (Thermo Fischer Scientific). Antibody information can be found in the “Antibodies” section of the “Methods” and in the “Reporting summary”.

### PFO* staining and FACS analysis

NIH3T3 (ATCC) or MEF cells were grown in 6-well plates and treated with the indicated conditions. Cells were lifted with 0.5% Triton–EDTA (Thermo Fischer Scientific) and gently pelleted by centrifugation at 200×*g* for 5 min. Pellets were washed gently two times with 1×PBS before incubation in blocking buffer (10 mg/mL BSA in 1×PBS) for 10 min on ice. Cells were pelleted once more before incubation with 5 μg/mL PFO* probe diluted in blocking buffer for 30 min on ice. Cells were gently washed one time with 1×PBS before analysis by FACS. Fluorescent intensity measurements by flow cytometry were performed on a SH800 Cell Sorter (Sony) using a 638 nm laser for excitation. Live cells were gated based on the forward scatter (FSC-A) and side scatter (SSC-A) plots and singlets were gated based on the forward scatter area (FSC-A) and height (FSC-H) in FlowJo v10 (FlowJo). No further gating was used to select cell populations. Outliers were identified using the identify outliers function in Prism 8 (GraphPad).

### Microscopy

Bright-field images were acquired on an Axio Imager.Z2 upright microscope (Zeiss) running AxioVision LE64, release 4.9.1 SP1 (Zeiss) for whole mount, in situ hybridization and *lacZ* staining. Immunofluorescence and PFO* images were acquired on either an Elipse Ti with a CSU-X1 spinning disc confocal (Nikon) and Clara interline CCD camera (Andor) with a Plan Apo ×60 oil objective (Nikon) running Nikon Elements 5.02 build 1266 (Nikon) or an LSM 800 confocal laser scanning microscope with a ×63 oil objective (Zeiss) running Zen 2 blue edition v1.0 (Zeiss). Cell length calculations, SMO intensity analysis, and PFO* intensity at the primary cilium analysis were done in FIJI.

### Recombinant protein expression and purification

CNPY4 constructs were synthesized by Genscript and subcloned into a pET28b plasmid with a 10xHis tag sequence. Cloning verification was done by DNA sequencing (Elim Biotechnology). Constructs were transformed into SHuffle T7 competent *E. coli* cells (New England Biolabs) and underwent antibiotic selection on Kanamycin plates for 16 h at 37 °C. A single colony was used to inoculate a Luria broth starter culture supplemented with Kanamycin for 16 h at 37 °C and 220 rpm shaking. 10 mL of starter culture was used to inoculate 900 mL of Terrific broth supplemented with Kanamycin and 100 mL of 10× phosphate buffer (0.17 M KH_2_PO_4_, 0.72 M K_2_HPO_4_). Cells were grown at 37 °C, 220 rpm shaking to an OD_600_ of 0.6–0.8 before being induced with isopropyl β-d-1-thiogalactopyranoside to a final concentration of 0.5 mM. Cultures were grown for an additional 20 h at 18 °C and 220 rpm shaking. Cells were collected by centrifugation with a JA 8.5 rotor (Avanti) at 4000×*g*, 4 °C for 40 min. Pellets were flash frozen for later purification or resuspended in binding buffer (50 mM HEPES, pH 8.0, 500 mM NaCl, 20 mM imidazole, pH 8.0, 5% glycerol) supplemented with DNaseI (Sigma Aldrich) and cOmplete mini EDTA-free protease inhibitor cocktail (Roche) and lysed via sonication at 30% amplitude, 4 s on, 2 s off, for a total of 5 min. Lysates were clarified in an Avanti centrifuge equipped with a JLA 25.50 rotor at 20,000×*g*, 40 min, 4 °C. Clarified lysates were incubated with Ni-NTA 6 Fast Flow beads (Cytiva Life Sciences), with gentle rotation, for 16 h at 4 °C before being applied to a gravity flow Econo-column (Bio-Rad). Beads were washed thoroughly with 20 column volumes of binding buffer followed by 10 column volumes of binding buffer supplemented with an additional 12.5 mM imidazole. The recombinant protein was eluted in 5 column volumes of elution buffer (binding buffer with 250 mM imidazole). The eluted protein was buffer exchanged back into low imidazole binding buffer and incubated with 1 mg of lab-purified, recombinant 3C protease for 16 h at 4 °C. Uncleaved protein was removed by passing the solution over fresh Ni-NTA 6 resin. The protein was then diluted 10 times with mono Q binding buffer (50 mM HEPES, pH 8.0) and applied to a MonoQ 5/50 GL column (Cytiva Life Sciences) connected to an AKTA Pure system (Cytiva Life Sciences) running UNICORN v6.4 (Cytiva Life Sciences). Recombinant hCNPY4ΔCt was eluted with a linear gradient of elution buffer (50 mM HEPES, pH 8.0, 500 mM NaCl). Collected elution fractions were pooled and concentrated using an Amicon Ultra-15 10k MWCO centrifugal filter (Merck Millipore) before being loaded onto a Superdex 200 16/600 column (Cytiva Life Sciences) equilibrated in size exclusion chromatography buffer (50 mM Bicine, pH 9.0, 150 mM NaCl). Fractions confirmed to contain pure hCNPY4ΔCt by SDS–PAGE analysis were pooled and flash-frozen in liquid nitrogen and stored at −80 °C.

### Circular dichroism

Purified CNPY proteins were analyzed on a J-810 spectropolarimeter (Jasco) running Spectra Manager for Windows 95/NT v1.27.00 (Jasco) at 1 nm steps. Proteins were analyzed at an approximate concentration of 2 μM in a 50 mM sodium phosphate buffer, pH 7.0 at 25 °C. Thermal melt data was collected at 222 nm with a temperature range of 25–95 °C in increments of 5 °C. hCNPY4ΔCt was additionally incubated with 30 μM of cholesterol, 20(S)-HC, or 24(S), 25-EC prior to thermal melt analysis for assessment of binding capacity. Data for three averaged reads was fitted using the log(agonist) vs. response–variable slope non-linear analysis in Prism 8 (GraphPad), and the LogEC50 from the analysis was reported as the melting temperature.

### Fluorescence polarization

Purified hCNPY4ΔCt in SEC buffer, as described above, were analyzed for binding to 50 nM BODIPY-cholesterol (Cayman Chemical) at the indicated protein concentrations. 1% Tween-20 was added to the reaction mixture. Experiments were performed with a reaction volume of 20 μL in quadruplicate using a black-bottom 384-well plates on an Analyst AD plate reader (Molecular Devices) running SoftMax pro v5.4.5 (Molecular Devices). Excitation and emission wavelengths used for the kinetic experiments were 480 and 508 nm, respectively, according to the manufacturer’s recommendation. Kinetic reads were performed over 15 min with 30 s intervals. As no significant difference was observed, signal was averaged across all time points with standard error calculated for each data point. Data was fitting using the Semilog line—*X* is log, *Y* is linear—non-linear analysis in Prism 8 (GraphPad).

### Co-immunoprecipitation

HEK293 cells (ATCC) were plated onto 6-cm plates and transfected the following day using lipofectamine 3000 (Thermo Fischer Scientific) according to the manufacturer’s protocol. Cells were lysed with lysis buffer (0.5% Triton X-100, 0.5% NP-40, 150 mM NaCl, 50 mM Tris pH8.0, 1 mM NaF, 1 mM Na(VO_4_)_3_, 1 mM EDTA, cOmplete mini EDTA-free protease inhibitor cocktail (Roche)) and immediately scraped. Cells were further lysed with rotation at 4 °C for 30 min followed by centrifugation for 5 min at 15,000 rpm. The clarified cell lysates were pre-cleared via incubation with Protein A beads (Thermo Fischer Scientific) for 30 min at 4 °C followed by centrifugation for 2 min at 14,000×*g*. The lysates were incubated with the indicated antibody for pull-down complexed to protein A beads overnight at 4 °C with rotation. Beads were washed three times with lysis buffer, and the bound proteins were eluted in SDS-loading buffer with boiling at 95 °C for 5 min.

### Mass-spectrometry-based sterolomics

NIH3T3 cells (ATCC) were transiently transfected with control or *Cnpy4* siRNA for 72 h, as described above, and cultured in low serum OptiMEM media (Thermo Fisher Scientific) for 48 h. Cells were pelleted and media supernatant collected for analysis. Quantification of sterol amounts were performed on by ultrahigh-performance liquid chromatography–tandem mass spectrometry using stable isotope-labeled internal standards, as previously described^82^. Data were analyzed using Analyst v.1.6.2 (AB Sciex). Amounts were normalized against the protein weight of each sample.

### Statistical analysis

All statistical analysis were performed using Prism 8 (GraphPad). Luciferase assay and qRT-PCR data were assumed to not be normally distributed, and significance were calculated using a Mann–Whitney non-parametric test. Cilia quantifications, FGF stimulation quantifications, PFO* FACS analysis, and mass-spectrometry-based sterolomics data were assumed to be normally distributed with nonequal variance between control and *Cnpy4* silenced cells, and significance were calculated using an unpaired Welch’s *t*-test. All statistical analyses were two-tailed.

### Reporting summary

Further information on research design is available in the Nature Research Reporting Summary linked to this article.

## Supplementary information

Supplementary Information

Peer Review File

Reporting Summary

## Data Availability

Raw data for all graphs and output from the mass spectrometry software are included in the source data file. Source data are provided with this paper. All additional information is available from the corresponding authors upon reasonable request.

## Source data

Source Data
