# Supplementary material for: CNPY4 inhibits the Hedgehog pathway by modulating membrane sterol lipids
Source: Nat Commun. 2022 May 3;13:2407. doi: 10.1038/s41467-022-30186-x (PMC9065090; doi:10.1038/s41467-022-30186-x)

CNPY4 inhibits the Hedgehog pathway by modulating membrane sterol lipids

Megan Lo, Amnon Sharir, Michael D. Paul, Hayarpi Torosyan, Christopher Agnew, Amy Li, Cynthia Neben, Pauline Marangoni, Libin Xu, David R. Raleigh, Natalia Jura, Ophir D. Klein

Supplementary Information

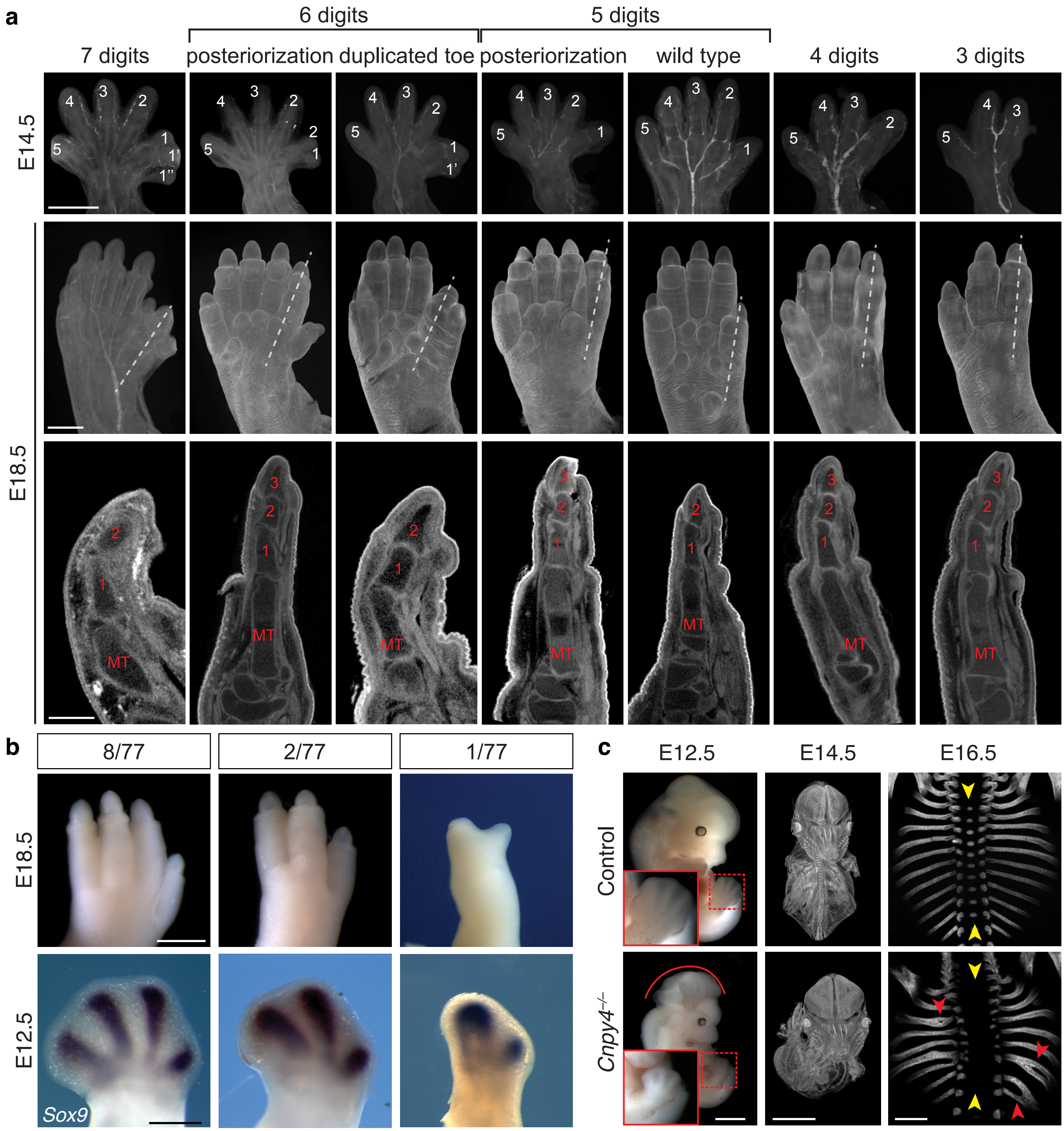

Supplementary Fig. 1 Developmental defects in a *Cnpy4* knockout mouse model. a Micro computed tomography (μCT) reconstructions of *Cnpy4* mutant limbs at embryonic day (E) 14.5 (top row) and E18.5 (bottom two rows) showing a range of hindlimb digit phenotypes from oligodactyly to polydactyly. Sagittal plane images through digit 1 (dashed line in middle row) showing the number of phalanges (bottom row). (MT) metacarpal. The scale bars represent 1 mm. **b** Dorsal view of *Cnpy4* mutant limbs at E18.5 (top) and whole mount *in situ* hybridization for *Sox9* at E12.5 (bottom) showing oligodactyly phenotypes. The scale bars represent 500 μm. **c** Whole mount images and μCT analysis showing (left) cranial neural tube defect (exencephaly); (middle) shortening and kinking of the body axis; (right) abnormal rib morphology, with fusions and bifurcations (red arrows). The scale bars represent 1 mm (left) and 2 mm (middle and right). All experiments were performed three independent times with similar results.

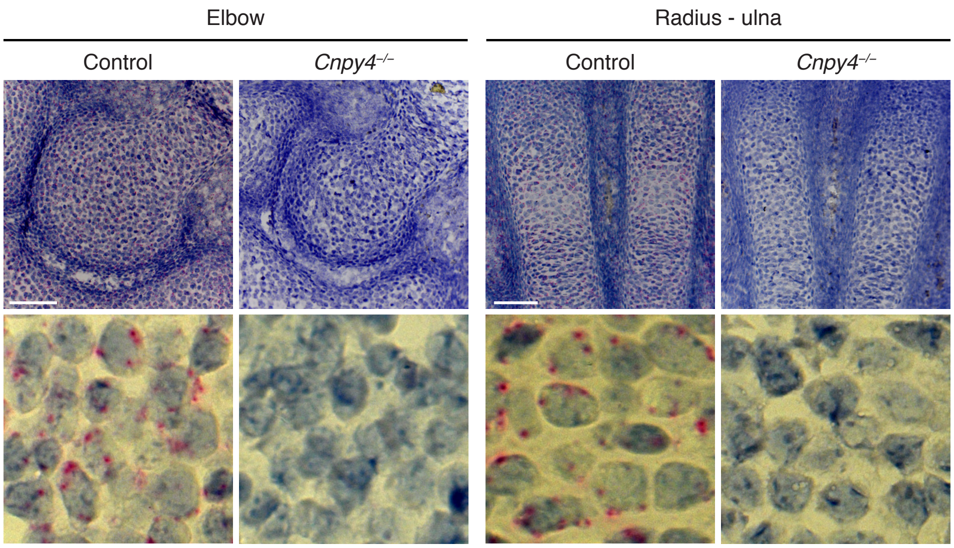

Supplementary Fig. 2 *Cnpy4* transcript expression in the long bones of the limbs. Expression pattern of *Cnpy4* transcripts in the elbow (left panels) and the radius - ulna (right panels) at embryonic day (E) 14.5. The scale bars represent 100 μm. Experiments were performed three independent times with similar results.

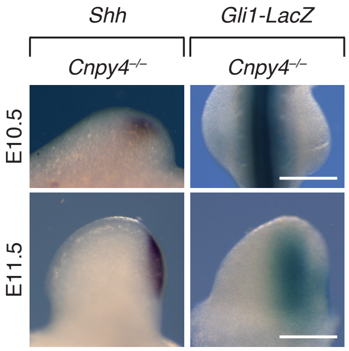

Supplementary Fig. 3 Reduction of *Shh* expression in minority of *Cnpy4* mutants. *In situ* hybridization of *Shh* (left) and *Gli1-LacZ* (right) in hindlimb buds at embryonic day (E) 10.5 and E11.5, showing reduced expression in a minority of *Cnpy4* mutants. The scale bars represent 500 μm. Experiments were performed three independent times with similar results.

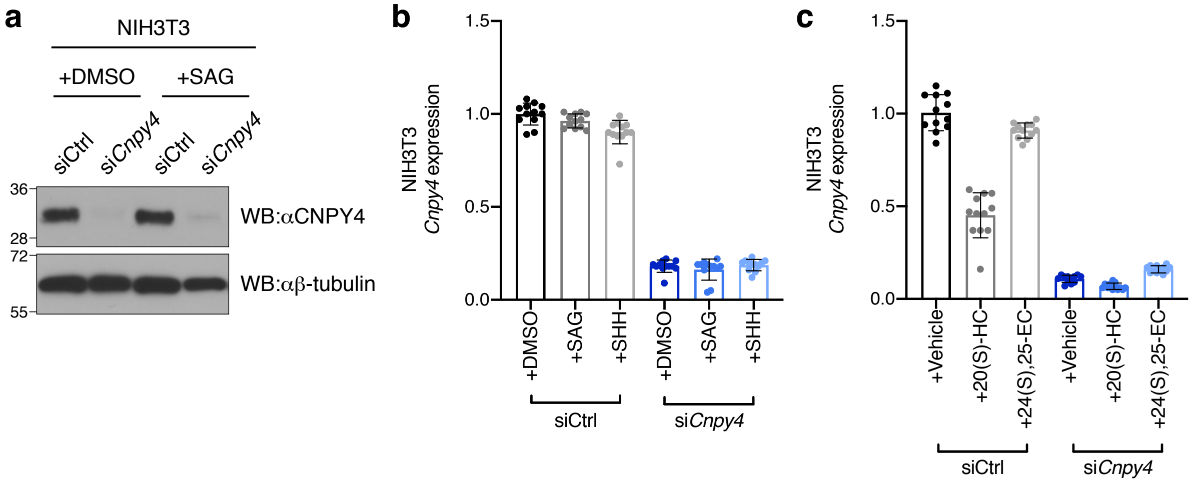

Supplementary Fig. 4 Knockdown of *Cnpy4* in NIH3T3 cells. a Protein levels in the lysates of NIH3T3 cells treated with control or *Cnpy4* siRNA were detected using the indicated antibodies by Western blot analysis. **b**, **c** qRT-PCR assessment of *Cnpy4* expression in ciliated NIH3T3 cells treated with control (grey bars) or *Cnpy4* (blue bars) siRNA and stimulated with SMO agonist (SAG), recombinant SHH (**b**), 20(S)-hydroxycholesterol (20(S)-HC), or 24(S), 25-epoxycholesterol (24(S),25-EC) (**c**). Data represent the mean ± SD (*n* = 12 from three biological and four technical replicates). All experiments were performed a minimum of three independent times with similar results.

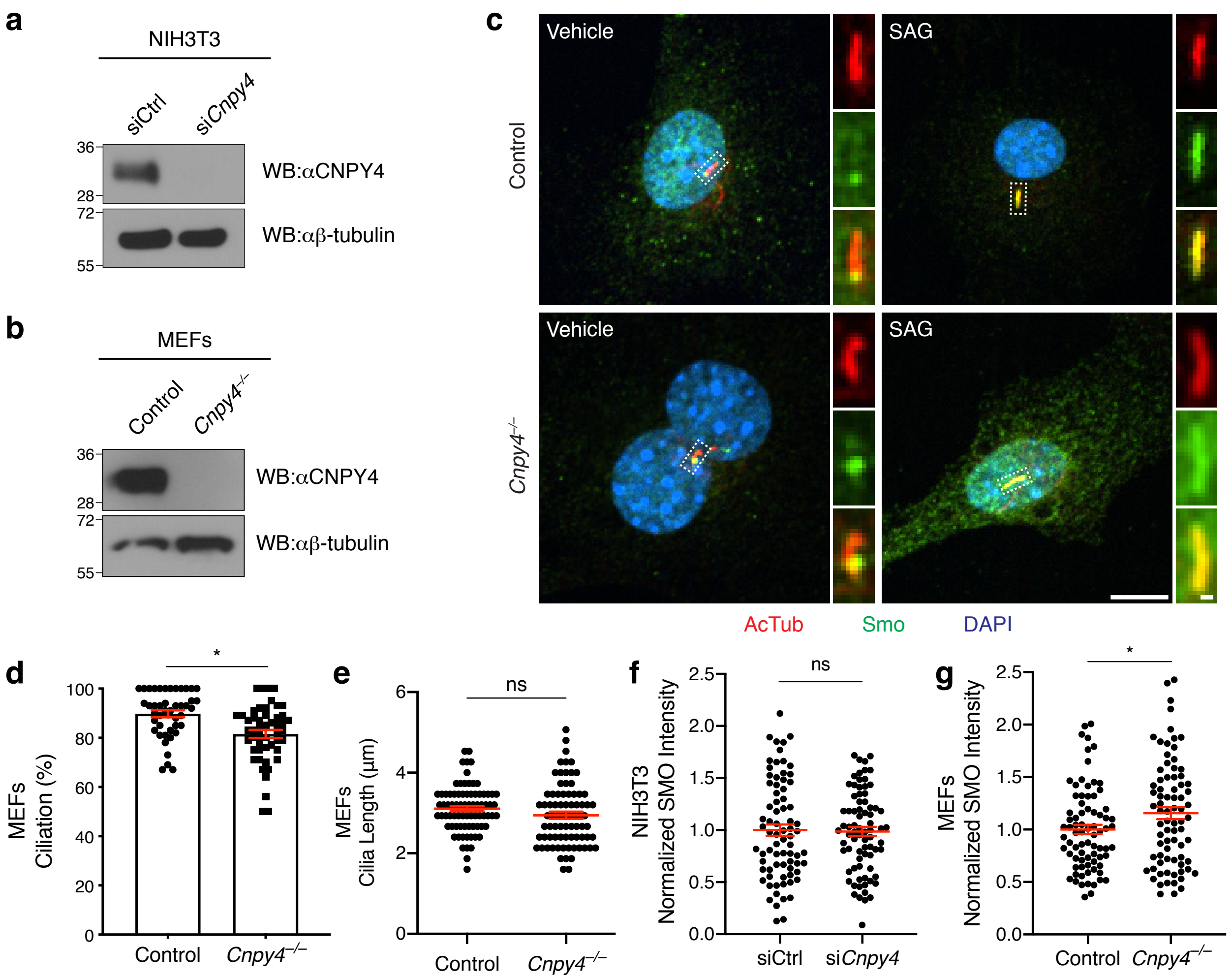

Supplementary Fig. 5 Loss of CNPY4 has a minor effect on the primary cilia. a, b Protein levels in the lysates of NIH3T3 cells treated with control or *Cnpy4* siRNA (**a**) or control and *Cnpy4*$^{-/-}$ MEF cells (**b**) used for immunofluorescence were detected using the indicated antibodies by Western blot analysis. Western blot analysis was performed a minimum of three independent times with similar results. **c** Immunofluorescence-based staining of primary cilia (acetylated tubulin, red), SMO (SMO, green), and nuclei (DAPI, blue) in control and *Cnpy4* null MEF cells with vehicle (DMSO) or Smo agonist (SAG) treatment. The scale bar represents 10 µm. Cilia scale bar represents 1 µm. **d** Quantification of the percentage of ciliated MEF cells, as assessed by acetylated tubulin immunofluorescence. Data represent the mean ± SEM (n = 717 control MEF cells and 676 *Cnpy4*$^{-/-}$ MEF cells from three independent experiments). Significance was calculated using a two-sided unpaired Welsh's t-test with *$p < 0.05$ ($p = 0.0322$). **e** Quantification of the length of cilia in MEF cells. Measurements were performed in FIJI using the acetylated tubulin channel. **f, g** Quantification of SMO trafficking to cilia in NIH3T3 (**f**) and MEF (**g**) cells. Analyses were done using FIJI by measuring the average fluorescence intensity of SMO over the length of the cilia in the appropriate channel. Average background fluorescence measured over the same length from NIH3T3 cell images were subtracted. Average background fluorescence in MEF cell images deviated by <5% and were not subtracted. Background-adjusted average fluorescence was then divided by the length of the cilia. Data were normalized to the average value of control siRNA treated or control cells. Data for **e**–**g** represent the mean ± SEM (n = 77 NIH3T3 cells from three biological replicates and n = 78 MEF cells from three independent experiments). Significance was calculated using a two-sided unpaired Welsh's t-test with ns $p > 0.05$ ($p_{\text{MEF cilia length}} = 0.1331$; $p_{\text{NIH3T3 Smo intensity}} = 0.8530$) and *$p < 0.05$ ($p_{\text{MEF Smo intensity}} = 0.0338$).

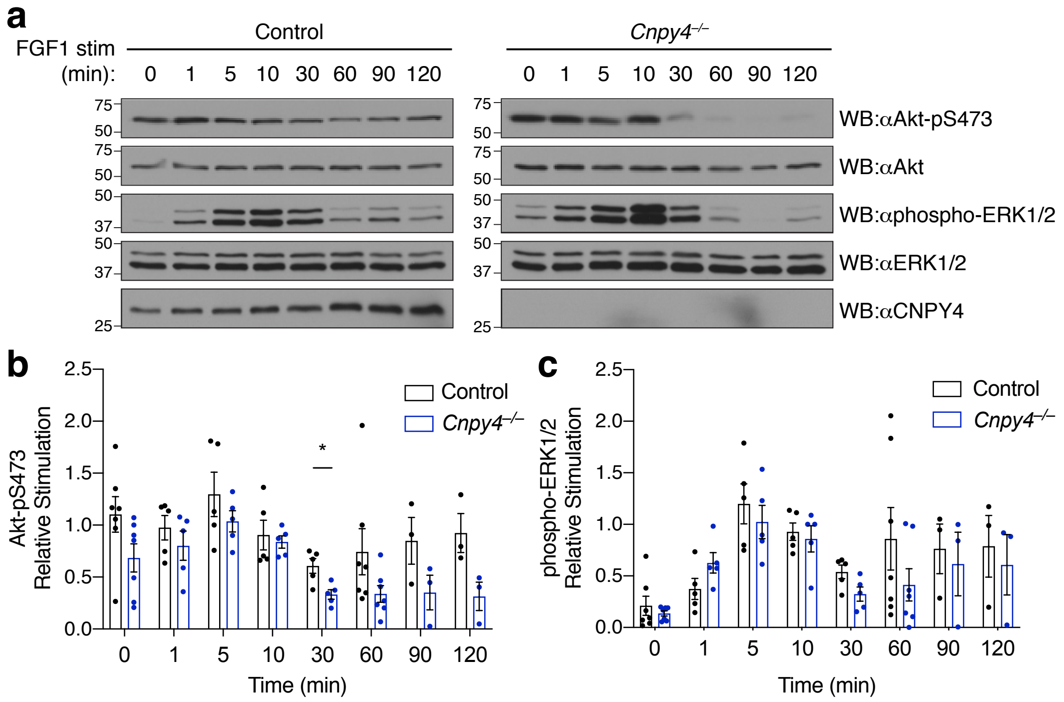

Supplementary Fig. 6 Absence of *Cnpy4* diminishes FGF signaling. a FGF1 stimulation of control and *Cnpy4*$^{-/-}$ MEF cells. Protein levels in lysates were normalized using a BCA assay and were detected using the indicated antibodies by Western blot analysis. **b**, **c** Quantifications of Akt-pS473 (**b**) and phospho-ERK1/2 (**c**) upon FGF1 stimulation of MEF cells. Data were doubly normalized against the corresponding non-phosphorylated species as a loading control and the highest value per experiment. Data represent the mean ± SEM ($n = 7$ for t = 0 and 60 minutes from seven independent experiments, $n = 5$ for t = 1, 5, 10, and 30 minutes from five independent experiments, and $n = 3$ for t = 90 and 120 minutes from three independent experiments). Significance was calculated using a two-sided unpaired Welch's t-test with $*p < 0.05$ ($p_{\text{Akt-pS473, t=30min}} = 0.0143$).

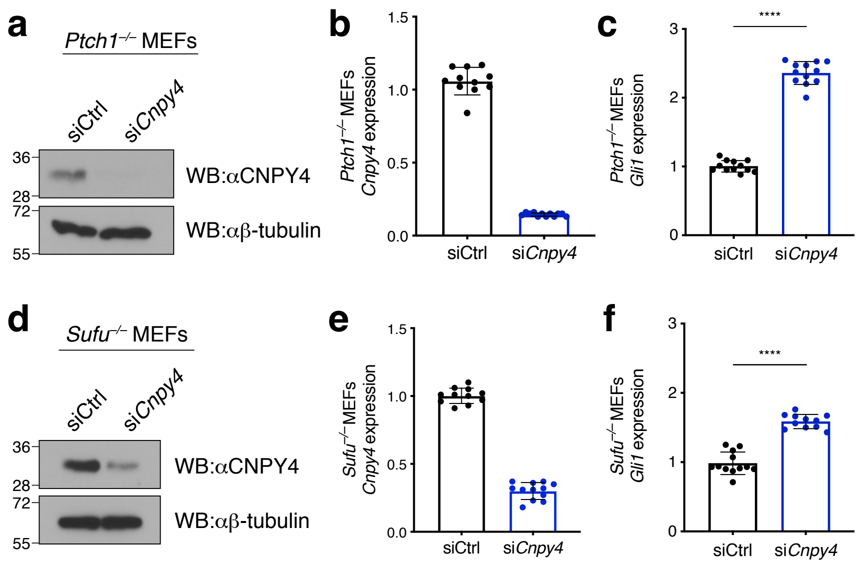

Supplementary Fig. 7 *Cnpy4* epistatic interaction with HH pathway components. a Protein levels in the lysates of *Ptch1*$^{-/-}$ MEF cells treated with control or *Cnpy4* siRNA were detected using the indicated antibodies by Western blot analysis. **b**, **c** qRT-PCR assessment of *Cnpy4* (**b**) and *Gli1* (**c**) expression in *Ptch1*$^{-/-}$ MEF cells treated with control (black bar) or *Cnpy4* (blue bar) siRNA. Data represent the mean ± SD ($n = 12$ from three biological and four technical replicates). Significance was calculated using a two-sided Mann-Whitney non-parametric test with ****$p < 0.0001$. **d** Protein levels in the lysates of *Sufu*$^{-/-}$ MEF cells treated with control or *Cnpy4* siRNA were detected using the indicated antibodies by Western blot analysis. **e**, **f** qRT-PCR assessment of *Cnpy4* (**e**) and *Gli1* (**f**) expression in *Sufu*$^{-/-}$ MEF cells treated with control (black bar) or *Cnpy4* (blue bar) siRNA. Data represent the mean ± SD ($n = 12$ from three biological and four technical replicates). Significance was calculated using a two-sided Mann-Whitney non-parametric test with ****$p < 0.0001$. All experiments were performed a minimum of three independent times with similar results.

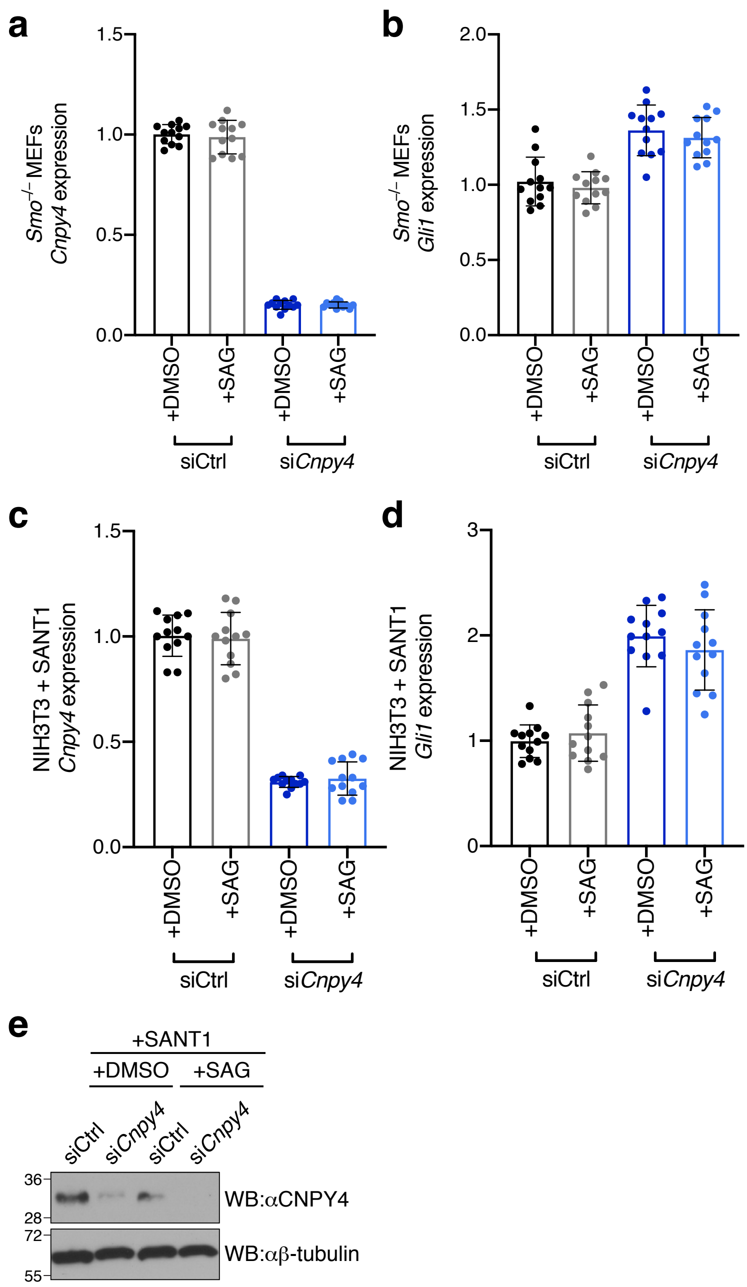

Supplementary Fig. 8 HH signal regulation by CNPY4 requires SMO. a, **b** qRT-PCR assessment of *Cnpy4* (**a**) or *Gli1* (**b**) expression in ciliated *Smo*$^{-/-}$ MEF cells treated with control (grey bars) or *Cnpy4* (blue bars) siRNA and stimulated with SMO agonist (SAG). Data represent the mean ± SD (*n* = 12 from three biological and four technical replicates. **c**, **d** qRT-PCR assessment of *Cnpy4* (**c**) or *Gli1* (**d**) expression in ciliated NIH3T3 cells treated with control (grey bars) or *Cnpy4* (blue bars) siRNA and stimulated with SAG and SMO antagonist (SANT-1). Data represent the mean ± SD (*n* = 12 from three biological and four technical replicates. **e** Protein levels in the lysates of NIH3T3 cells treated with control or *Cnpy4* siRNA and stimulated with SAG and SANT-1 were detected using the indicated antibodies by Western blot analysis. All experiments were performed a minimum of three independent times with similar results.

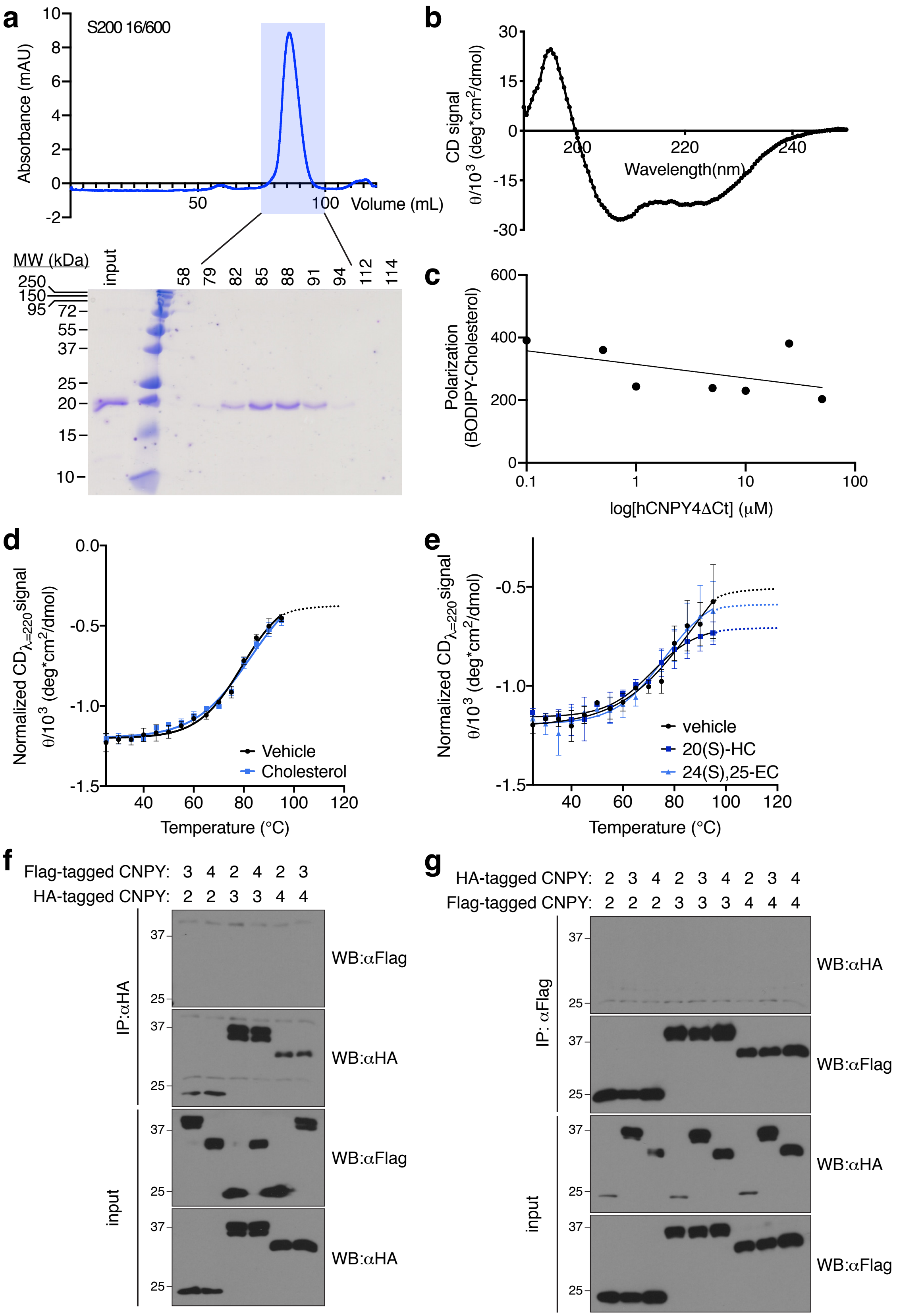

Supplementary Fig. 9 Recombinant CNPY4 does not bind sterols involved in HH activation. **a** Size exclusion chromatography profile for the purification of a construct of hCNPY4 lacking its C-terminal tail (hCNPY4ΔCt) and the accompanying Coomassie-stained gel. **b** Circular dichroism (CD) of hCNPY4ΔCt at room temperature. **c** Fluorescence polarization of a BODIPY-cholesterol probe incubated with increasing concentrations of purified hCNPY4ΔCt. Data represent the mean ± SEM (n = 92 from 4 biological and 23 technical replicates). **d**, **e** Thermal melt of hCNPY4ΔCt incubated with vehicle (chloroform) or cholesterol (**d**)

or vehicle (methyl-β-cyclodextrin), 20(S)-hydroxycholesterol (20(S)-HC), or 24(S), 25-epoxycholesterol (24(S),25-EC) (**e**) analyzed by CD at a wavelength of 222 nm. Data were normalized to the average value per condition for each experiment. Data represent the mean ± SEM (n = 3 independent experiments). **f**, **g** Co-immunoprecipitation of Flag-tagged and HA-tagged variants of wild-type CNPY2, CNPY3, and CNPY4 for homo- and hetero-dimerization. Proteins were transiently expressed in HEK293 cells, pulled-down using either an anti-HA (**f**) or an anti-Flag (**g**) antibody. Protein levels were detected with the indicated antibodies by Western blot analysis. Co-immunprecipitation experiments were performed a minimum of three independent times with similar results.

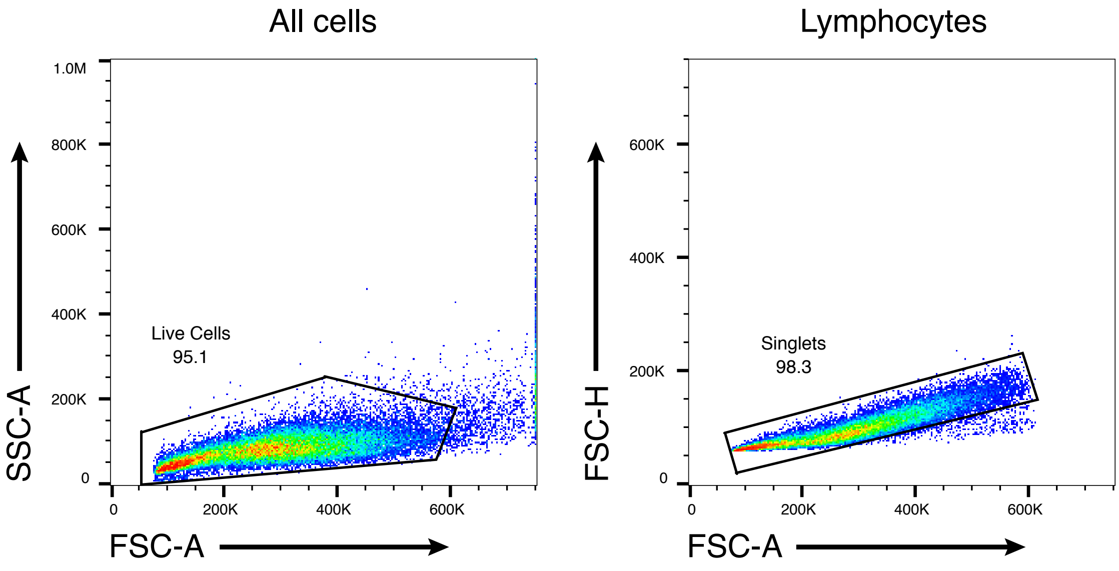

Supplementary Fig. 10 Gating strategy for PFO* FACS analysis. SSC-A vs. FSC-A plot showing gating for live cell population (left) and FSC-H vs FSC-A plot showing gating for singlet population (right) used for the FACS analysis on PFO* staining.

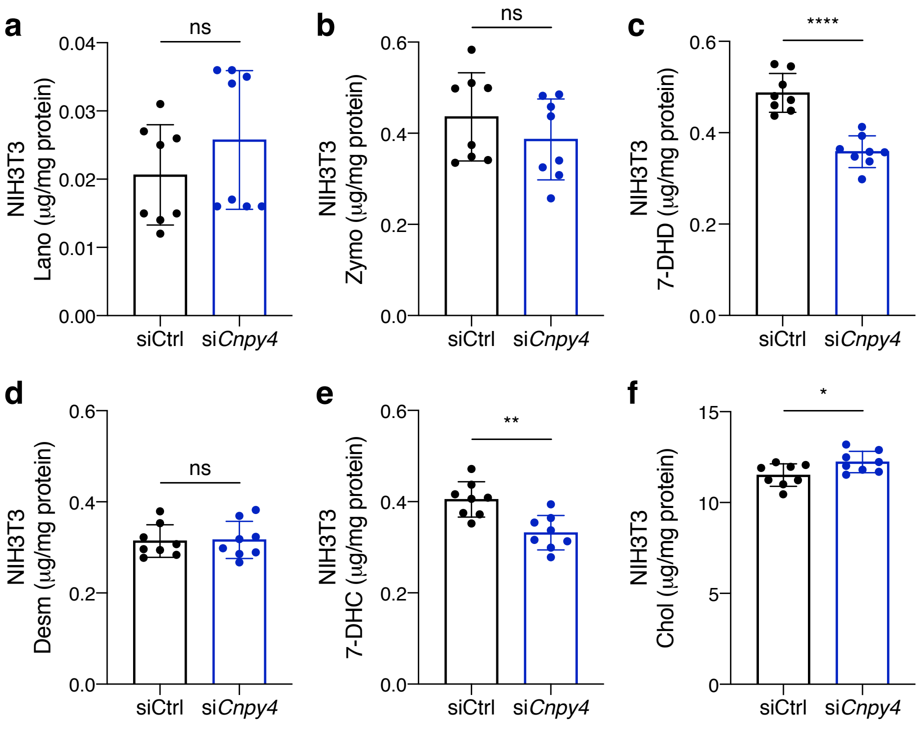

Supplementary Fig. 11 Effect of *Cnpy4* knockdown on the levels of sterol precursors in cholesterol biosynthesis. a–f Mass spectrometry-based sterolomics analysis of the cell lysates from NIH3T3 cells treated with control (black bars) or *Cnpy4* (blue bars) siRNA, showing levels of cholesterol precursors lanosterol (Lano) (**a**), zymosterol (Zymo) (**b**), 7-dehydrodesmosterol (7-DHD) (**c**), desmosterol (Desm) (**d**), and 7-dehydrocholesterol (7-DHC) (**e**), and cholesterol (Chol) (**f**). Data represent the mean ± SD (n = 8 replicates). Significance was calculated using a two-sided unpaired Welsh's t-test with ns $p > 0.05$ ($p_{\text{Lano}} = 0.2695$; $p_{\text{Zymo}} = 0.3037$; $p_{\text{Desm}} = 0.8977$), *$p < 0.05$ ($p_{\text{Chol}} = 0.0315$), **$p < 0.005$ ($p_{\text{7-DHC}} = 0.0019$), ****$p < 0.0001$.

Supplementary Table 1 qRT-PCR primers.

**Gene**	**Forward (5’ – 3’)**	**Reverse (5’ – 3’)**
Gapdh (mouse)	tgcccccatgtttgtgatg	tgtggtcatgagcccttcc
Cnpy4 (mouse)	gacaaaagaggaggaagatgacacag	ccaggatccgctcgcacaaattctcc
Gli1 (mouse)	ggtgctgcctatagccagtgtcctc	gtgccaatccggtggagtcagaccc

Uncropped Western blots

Figure 1c

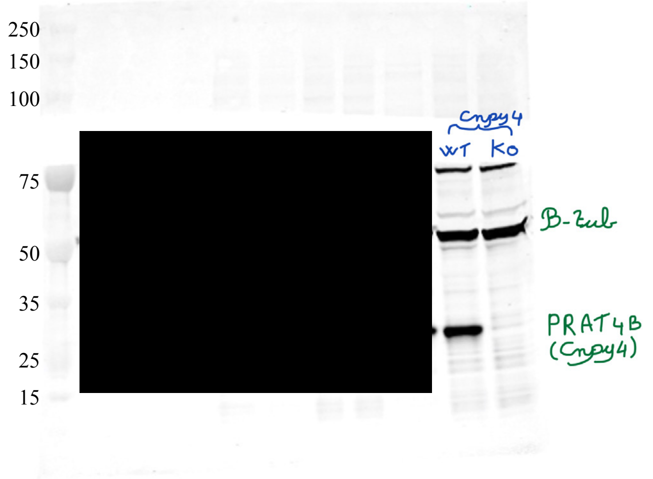

Green (800) channel, intensity 5.0
Protein amount for each isomer is about 20ug.

Supplementary Figure 5a

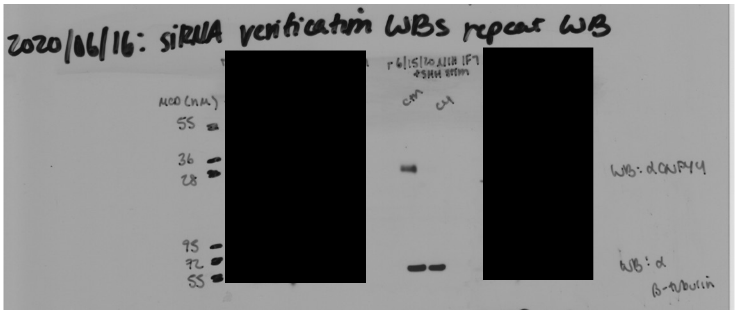

Supplementary Figure 5b

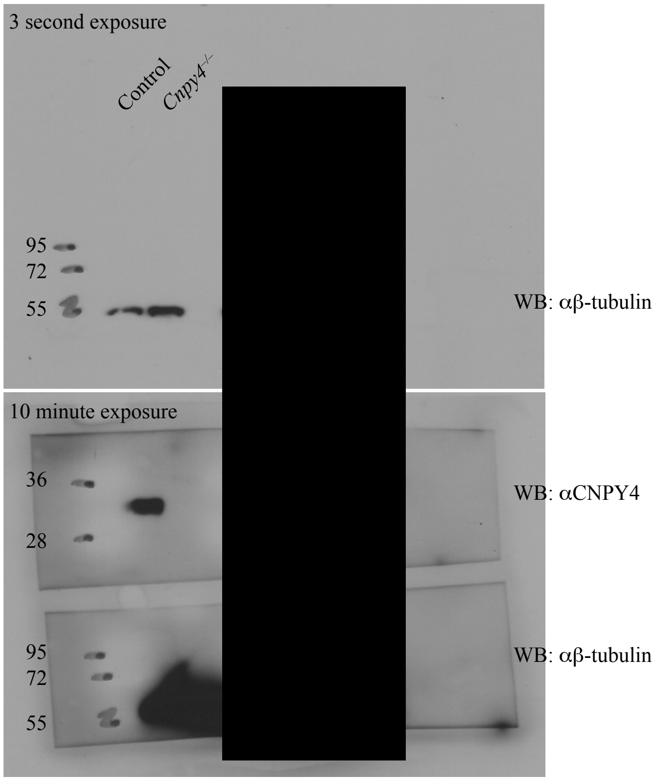

Supplementary Figure 8e

Supplementary Figure 4a

Supplementary Figure 7a, d

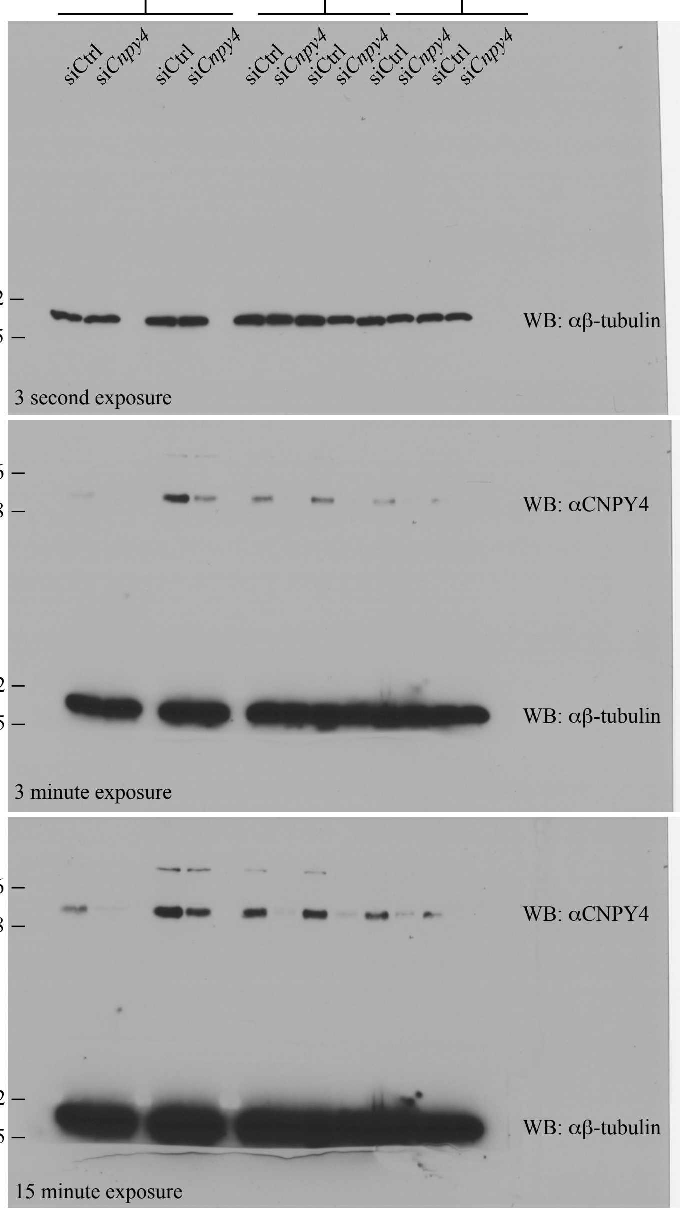

Uncropped Western blots (cont.)

Supplementary Figure 6a

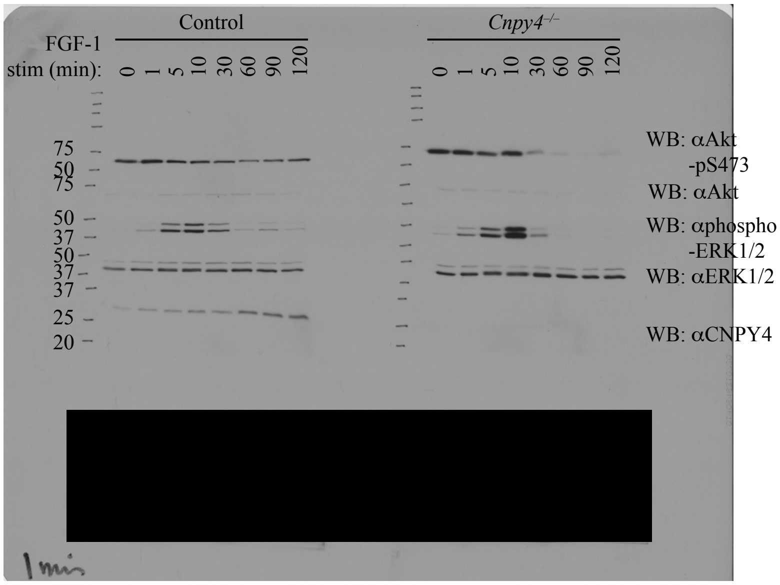

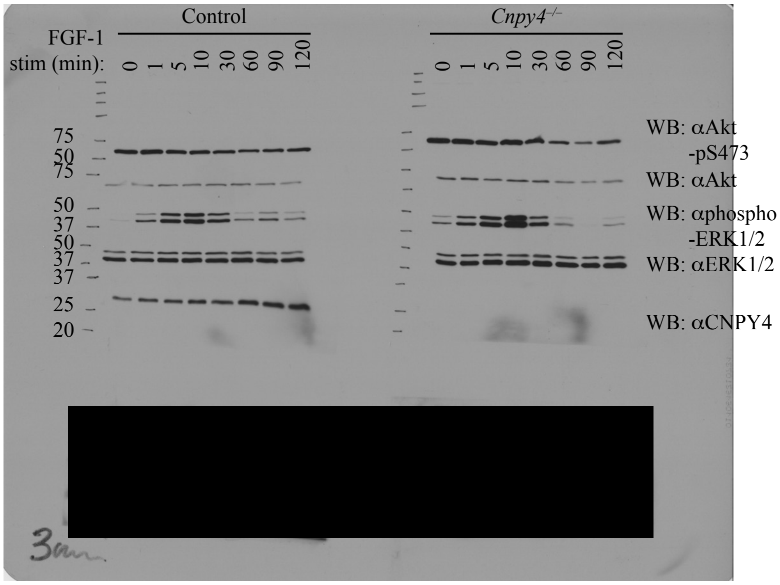

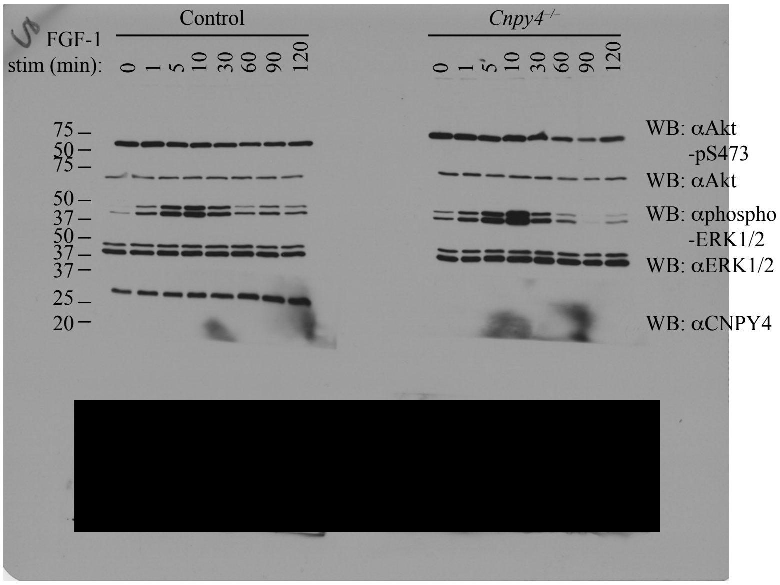

Supplementary Figure 9f

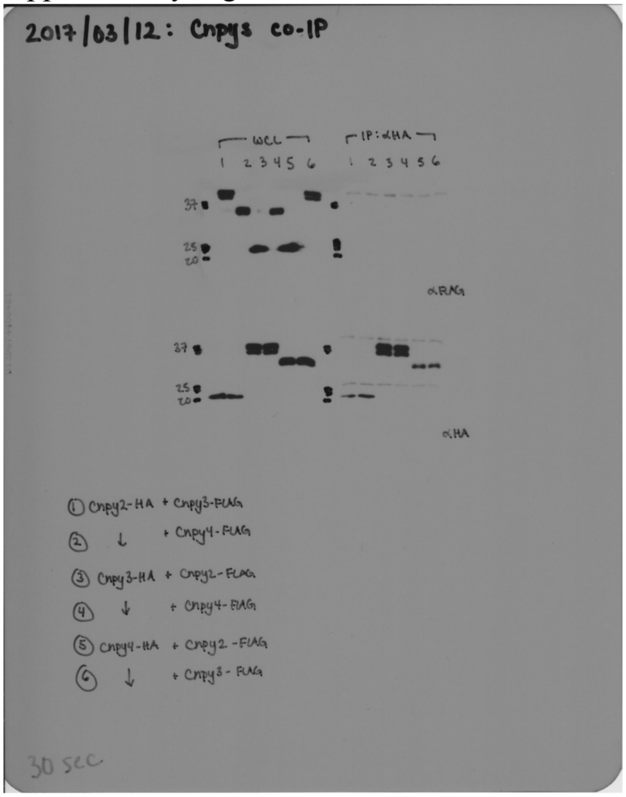

Supplementary Figure 9g

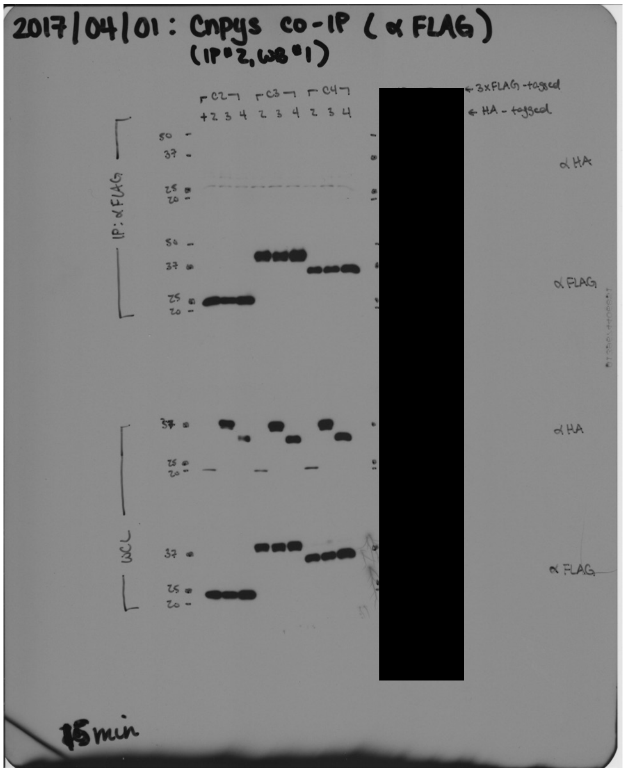

Supplement: Supplementary file 1 — Supplementary Information [file 41467_2022_30186_MOESM1_ESM.pdf]
