# Supplementary material for: CNPY4 inhibits the Hedgehog pathway by modulating membrane sterol lipids
Source: Nat Commun. 2022 May 3;13:2407. doi: 10.1038/s41467-022-30186-x (PMC9065090; doi:10.1038/s41467-022-30186-x)
Supplement: Supplementary file 3 — Reporting Summary [file 41467_2022_30186_MOESM3_ESM.pdf]

nature portfolio

Corresponding author(s): Ophir D. Klein and Natalia Jura

Last updated by author(s): Mar 17, 2022

Reporting Summary

Nature Portfolio wishes to improve the reproducibility of the work that we publish. This form provides structure for consistency and transparency in reporting. For further information on Nature Portfolio policies, see our Editorial Policies and the Editorial Policy Checklist.

Statistics

For all statistical analyses, confirm that the following items are present in the figure legend, table legend, main text, or Methods section.

n/a	Confirmed	
☐	☒	The exact sample size (n) for each experimental group/condition, given as a discrete number and unit of measurement
☐	☒	A statement on whether measurements were taken from distinct samples or whether the same sample was measured repeatedly
☐	☒	The statistical test(s) used AND whether they are one- or two-sided<br>*Only common tests should be described solely by name; describe more complex techniques in the Methods section.*
☒	☐	A description of all covariates tested
☐	☒	A description of any assumptions or corrections, such as tests of normality and adjustment for multiple comparisons
☐	☒	A full description of the statistical parameters including central tendency (e.g. means) or other basic estimates (e.g. regression coefficient) AND variation (e.g. standard deviation) or associated estimates of uncertainty (e.g. confidence intervals)
☐	☒	For null hypothesis testing, the test statistic (e.g. F, t, r) with confidence intervals, effect sizes, degrees of freedom and P value noted<br>*Give P values as exact values whenever suitable.*
☒	☐	For Bayesian analysis, information on the choice of priors and Markov chain Monte Carlo settings
☒	☐	For hierarchical and complex designs, identification of the appropriate level for tests and full reporting of outcomes
☒	☐	Estimates of effect sizes (e.g. Cohen's d, Pearson's r), indicating how they were calculated

Our web collection on statistics for biologists contains articles on many of the points above.

Software and code

Policy information about availability of computer code

Data collection	Avizo Lite v9.1.1 (Thermo Fischer Scientific); AxioVision LE64, release 4.9.1 SP1 (Zeiss); Nikon Elements 5.02 build 1266 (Nikon); Zen 2 blue edition v1.0 (Zeiss); UNICORN v6.4 (Cytiva Life Sciences); Spectra Manager for Windows 95/NT v1.27.00 (Jasco); SoftMax pro v5.4.5 (Molecular Devices); Analyst v.1.6.2 (AB Sciex)
Data analysis	Back projection filtered algorithm (Zeiss); Identify Outliers (GraphPad); log(agonist) vs. response–Variable slope non-linear analysis (GraphPad); Semilog line–X is log, Y is linear non-linear analysis (GraphPad); Prism 8 (GraphPad); FIJI; FlowJo v10; Analyst v.1.6.2 (AB Sciex)

For manuscripts utilizing custom algorithms or software that are central to the research but not yet described in published literature, software must be made available to editors and reviewers. We strongly encourage code deposition in a community repository (e.g. GitHub). See the Nature Portfolio guidelines for submitting code & software for further information.

Data

Policy information about availability of data

All manuscripts must include a data availability statement. This statement should provide the following information, where applicable:

- Accession codes, unique identifiers, or web links for publicly available datasets
- A description of any restrictions on data availability
- For clinical datasets or third party data, please ensure that the statement adheres to our policy

Raw data for all graphs and output from the mass spectrometry software are included in the source data file. Source data are provided with this paper. All additional information is available from the corresponding authors upon reasonable request.

Field-specific reporting

Please select the one below that is the best fit for your research. If you are not sure, read the appropriate sections before making your selection.

☒ Life sciences ☐ Behavioural & social sciences ☐ Ecological, evolutionary & environmental sciences

For a reference copy of the document with all sections, see nature.com/documents/nr-reporting-summary-flat.pdf

Life sciences study design

All studies must disclose on these points even when the disclosure is negative.

Sample size	Sample size was based on previously published studies of similar design (Raleigh et al., 2018). Sample size information is noted in the accompanying figure legends.
Data exclusions	Data was excluded where indicated in the source data due to either instrument error or where one technical replicate varied by more than 3 standard deviations from the other technical replicates. Excluded data is noted in the source data file. Outliers were excluded in the FACS analysis on PFO* staining using the "Identify Outliers" function on Prism 8, in accordance to previously published studies of similar design (Kinnebrew et al., 2019).
Replication	All in vivo, biochemical, and protein-based experiments were repeated a minimum of 3 successful replicates and a sentence stating so has been added to the accompanying figure legends.
Randomization	The experiments used in the study did not require randomization.
Blinding	The experiments used in the study did not require blinding.

Reporting for specific materials, systems and methods

We require information from authors about some types of materials, experimental systems and methods used in many studies. Here, indicate whether each material, system or method listed is relevant to your study. If you are not sure if a list item applies to your research, read the appropriate section before selecting a response.

Materials & experimental systems

n/a	Involved in the study	
☐	☒	Antibodies
☐	☒	Eukaryotic cell lines
☒	☐	Palaeontology and archaeology
☐	☒	Animals and other organisms
☒	☐	Human research participants
☒	☐	Clinical data
☒	☐	Dual use research of concern

Methods

n/a	Involved in the study	
☒	☐	ChIP-seq
☐	☒	Flow cytometry
☒	☐	MRI-based neuroimaging

Antibodies

Antibodies used	The following primary antibodies were used in this study: beta-tubulin 1:2000 for WB (#2128, rabbit; Cell Signaling Technology); PRAT4B/CNPY4 1:500 for WB (AF5015, goat; R&D Systems); acetylated tubulin 1:1000 for IF (T6793, mouse, clone 6-11B-1; MilliporeSigma); Smoothened 1:1000 for IF (ab80683, rabbit; Abcam); Golgin97 1:100 for IF (A-21270, mouse, clone CDF4; Thermo Fischer Scientific); PDI 1:500 for IF (NB300-517, mouse, clone RL90; Novus Biologicals); TOM70 1:500 for IF (sc-17764, mouse, clone F-10; Santa Cruz Biotechnology); Akt-pS473 1:2000 for WB (#9271, rabbit; Cell Signaling Technology); Akt 1:2000 for WB (#9272, rabbit; Cell Signaling Technology); phospho-ERK1/2 1:2000 for WB (#9101, rabbit; Cell Signaling Technology); ERK1/2 1:2000 for WB(#9102, rabbit; Cell Signaling Technology); FLAG 1:2000 for WB (F1804, mouse, clone M2; MilliporeSigma); FLAG 1:500 for IF, 1:1000 for WB (#2368, rabbit; Cell Signaling Technology); and HA 1:500 for WB (sc-7392, mouse, clone F-7; Santa Cruz Biotechnology).<br>The following fluorescently-labeled secondary antibodies were used in this study: Goat anti-Mouse IgG (H+L) Cross-Adsorbed Secondary Antibody, Alexa Fluor 647 1:500 for IF (A-21235; Thermo Fischer Scientific); Goat anti-Rabbit IgG (H+L) Highly Cross-Adsorbed Secondary Antibody, Alexa Fluor Plus 488 1:500 for IF (A32731; Thermo Fischer Scientific); and Goat anti-Mouse IgG (H+L) Cross-Adsorbed Secondary Antibody, Alexa Fluor 568 1:500 for IF (A-11004; Thermo Fischer Scientific).<br>The following secondary antibodies were used in this study: Veriblot for IP detection reagent (HRP) 1:2000 for WB (ab131366; Abcam); Anti rabbit IgG, HRP-linked Antibody 1:5000 for WB (#7074; Cell Signaling Technology); and Cytiva's Amersham ECL Mouse IgG, HRP-linked whole Ab 1:5000 for WB (from sheep) (NA931; Cytiva Life Sciences).
Validation	The HA and both FLAG antibodies were validated through empty vector transfection. The PRAT4B/CNPY4 antibody was validated through knockout and siRNA knockdown followed by Western blotting (loss of band of expected size). The beta-tubulin, acetylated

tubulin, Smoothened, Golgin97, PDI, TOM70, Akt-PS473, Akt, phospho-ERK1/2, and ERK1/2 antibodies were validated through the manufacturer. Secondary antibodies were validated through no primary antibody controls.

Eukaryotic cell lines

Policy information about cell lines

Cell line source(s)	HEK293 (American Type Culture Collection [ATCC]); COS7 (ATCC); NIH3T3 (gifts from Jeremy Reiter (UCSF) originally purchased from ATCC); WT, Smo–/–, and Cnpy4–/– MEF cell lines (self-generated); Sufu–/– and Ptch1–/– (gifts from Pao-Tien Chuang (UCSF) and Jeremy Reiter (UCSF))
Authentication	Cell lines were not authenticated.
Mycoplasma contamination	All cell lines were tested quarterly for mycoplasma contamination using the MycoAlert mycoplasma detection kit (Lonza) and tested negative.
Commonly misidentified lines (See ICLAC register)	No commonly misidentified cell lines were used in the study.

Animals and other organisms

Policy information about studies involving animals; ARRIVE guidelines recommended for reporting animal research

Laboratory animals	Species: Mice<br>Strain: Cnpy4–/– 129-C57BL/6J hybrid mice and Cnpy4–/–;Gli1LacZ mice on a mixed background<br>Sex: Male and female<br>Age: Embryonic days 10.5, 11.5, 12.5, 14.5, 16.5, and 18.5
Wild animals	Study did not involve wild animals.
Field-collected samples	Study did not involve samples collected from the field.
Ethics oversight	Mice were maintained in the University of California, San Francisco (UCSF) specific pathogen-free animal facility in accordance with the guidelines established by the Institutional Animal Care and Use Committee (IACUC) and Laboratory Animal Resource Center (LARC). All experimental procedures were approved by the LARC at UCSF.

Note that full information on the approval of the study protocol must also be provided in the manuscript.

Flow Cytometry

Plots

Confirm that:

☒ The axis labels state the marker and fluorochrome used (e.g. CD4-FITC).

☒ The axis scales are clearly visible. Include numbers along axes only for bottom left plot of group (a 'group' is an analysis of identical markers).

☒ All plots are contour plots with outliers or pseudocolor plots.

☒ A numerical value for number of cells or percentage (with statistics) is provided.

Methodology

Sample preparation	Cells were grown in 6-wells and treated with indicated conditions. Cells were lifted with 0.5% Triton-EDTA and gently pelleted by centrifugation at 200xg for 5 minutes. Pellets were washed gently two times with 1x PBS before incubation in blocking buffer (10 mg/mL BSA in 1x PBS) for 10 minutes on ice. Cells were pelleted once more before incubation with 5 µg/mL PFO* probe diluted in blocking buffer for 30 minutes on ice. Cells were gently washed one time with 1x PBS before analysis by FACS.
Instrument	Sony Cell Sorter SH800
Software	FlowJo
Cell population abundance	N=92183 for control MEFs and N=74848 for Cnpy4–/– MEFs; N=244550 for siCtrl NIH3T3 cells and N=291375 for siCnpy4 NIH3T3 cells
Gating strategy	Fluorescent intensity measurements by flow cytometry were performed on a SH800 Cell Sorter (Sony) using a 638 nm laser for excitation. Live cells were gated for based on the forward scatter (FSC-A) and side scatter (SSC-A) plots and singlets were gated for based on the forward scatter area (FSC-A) and height (FSC-H) FlowJo v10 (FlowJo).

☒ Tick this box to confirm that a figure exemplifying the gating strategy is provided in the Supplementary Information.
