# Supplementary material for: CNPY4 inhibits the Hedgehog pathway by modulating membrane sterol lipids
Source: Nat Commun. 2022 May 3;13:2407. doi: 10.1038/s41467-022-30186-x (PMC9065090; doi:10.1038/s41467-022-30186-x)
Supplement: Supplementary file 2 — Peer Review File [file 41467_2022_30186_MOESM2_ESM.pdf]

nature portfolio

Peer Review File

CNPY4 inhibits the Hedgehog pathway by modulating membrane sterol lipids

Open Access This file is licensed under a Creative Commons Attribution 4.0 International License, which permits use, sharing, adaptation, distribution and reproduction in any medium or format, as long as you give appropriate credit to the original author(s) and the source, provide a link to the Creative Commons license, and indicate if changes were made. In the cases where the authors are anonymous, such as is the case for the reports of anonymous peer reviewers, author attribution should be to 'Anonymous Referee' followed by a clear attribution to the source work. The images or other third party material in this file are included in the article's Creative Commons license, unless indicated otherwise in a credit line to the material. If material is not included in the article's Creative Commons license and your intended use is not permitted by statutory regulation or exceeds the permitted use, you will need to obtain permission directly from the copyright holder. To view a copy of this license, visit http://creativecommons.org/licenses/by/4.0/.

REVIEWER COMMENTS</B>

Reviewer #1 (Remarks to the Author):

The manuscript by Lo et al. focuses on the mechanistic activities Cnpy4, a novel regulator of the Hh pathway. In addition to using a murine transgenic line to demonstrate characteristic Hh related phenotypes including polydactytly, the authors identified Cnyp4 functions at the level of Smo to elevate sterol lipid composition of the ciliary membrane. As several recent studies have addressed the role of membrane lipid composition in terms of pathway modulation, this work would be of great interest to the Hedgehog community. However, there seem to be a few missed opportunities, that if addressed, could complete the story and increase the impact and biomedical relevance of the study.

1. In general, the Introduction does not set up the rationale for the study very well. There is background on the Hh transduction mechanism, but not on why examining this family member is important. Considering the link between cholesterol, Hh, and therapeutics that are widely available to reduce cholesterol levels, a more biomedically relevant introduction would increase the significance of the work.

2. Where is the Cnpy4 transcript expressed throughout the embryo (temporally/spatially)? The endogenous expression pattern of Cnpy4 (or other family members) is not shown. Perhaps data from extended data figure 8, should also be added into main figures to partially address these questions. These data are important to support the conclusions made by authors.

3. Previous reports have linked Cnpy genes to Fgf signaling. Is Fgf signaling maintained in Cnpy4-/- mutants? This seems highly relevant considering the interactions between FGF and SHH, and Gli3/Fgf during limb development.

4. Was genetic background of mice considered in regard to the variable phenotype of Cnpy4-/- mice? There is precedence for variable expression of ciliary genes, and this can be observed on different backgrounds. Furthermore, this could offer an alternative hypothesis of genetic modifiers impacting expression and eventual phenotype.

5. Onset of the polydactylous phenotypes, especially those in ciliary mutants, has been linked to a loss of the Gli3 repressor. Is the Gli3R isoform lost in Cnpy4-/- mutants?

6. While it is understandable why the mechanistic studies were carried out under in vitro conditions, there is no attempt to test the suggested mechanism in vivo. For example, can the hyperactivity of the Hh pathway and subsequent Hh-related phenotypes in Cnpy4-/- mice be mitigated by reducing cholesterol production genetically or pharmacologically?

Reviewer #2 (Remarks to the Author):

Lo et al. report CNPY4 functions in the regulation of the Hh pathway. They demonstrate that Cnpy4 KO mice showed polydactyly, which is typically observed in Hh-misregulated animals. In KO mice and siRNA-mediated knockdown cells, Hh hyperactivation is also confirmed. Although the downregulation of Cnpy4 does not affect general ciliary morphology and trafficking, they found that cellular sterol level is abnormally upregulated in Cnpy4-knockdown condition. In general, the manuscript is written and presented well, and features logical experimental designs (except Fig. 4).

The main criticism would be that, while the authors found a striking Hh-misregulated phenotype in knockout mice, the exact molecular mechanism of Cnpy4 for Hh signaling is still poorly characterized. Accordingly, this reviewer thinks that Cnpy4 is simply one of the dozens of genes involved in the HH pathway. It also does not make a great deal of sense that Cnpy4 as a lipid binding protein plays a role on lipids without direct interaction. In short, the effect of Cnpy4 KO/KD on sterol regulation should be investigated more deeply. For example, whether Cnpy4 downregulates general cholesterol level and/or targeting of cholesterol into specific organelles should be clarified. In addition, the following experiments are suggested to be performed to support the current dataset shown in Fig. 4 a-c.

1. Quantification of PFO at primary cilia in WT and KD cells

2. PFO staining in WT and KO embryos

3. PFO staining with Cnpy4 overexpression in WT and KD cells

4. Depletion of cholesterol (for example by beta-cyclodextrin) in KD cells to see if HH activity goes back to normal level (functional rescue to confirm the functional specificity of Cnpy4)

Other miscellaneous points:

1. Introduction should clarify what is already known about Cnpy4.

2. The molecular mechanism of Cnpy4-mediated Hh signaling should be examined in more detail. It seems that Cnpy4 inhibition does not affect Smo entry upon Hh ligands. The level of Smo activation and Sufu inactivation should be compared in WT and KD cells.

3. Line 206: possible typo “it possible that”

Reviewer #3 (Remarks to the Author):

This study by Lo et al identifies Canopy 4 (CNPY4) as a regulator of the Hedgehog signaling pathway. This is an interesting and unexpected finding that should be published, however there are some issues.

As described in the Abstract, the paper has two aspects, one of which is characterizing CNPY4 knockout animals in terms of Hedgehog pathway signaling, and the other of which is a speculative proposal for how CNPY4 may be acting through modulating membrane cholesterol levels. The first aspect is well-developed and the data are convincing, I need no further convincing that CNPY4 is important for Hedgehog signaling through SMO and for proper digit development! However, the second aspect is too speculative and should either be toned down or more work needs to be done. There are several points of concern:

1) The Abstract states (and it is repeated throughout the paper) that knockdown of CNPY4 “elevates membrane levels of accessible… cholesterol”. All of this is based on one set of sub-figures in Fig. 4a-c using a probe for plasma membrane accessible cholesterol. Much more would need to be done to refine this correlation. Does overall cholesterol increase or is it just the pool of accessible cholesterol?

2) In Figure 4D, the authors draw CNPY4 in the ER and imply that it modulates cholesterol synthesis. There is no data for this. Does CNPY4 affect the activation of SREBP transcription factors and through that affect cholesterol synthesis? Or does it activate some transport mechanism between the ER and plasma membrane?

I think keeping the focus of the paper on the remarkable effect of CNPY4 knockdown on digit development and merely pointing out a correlation with accessible cholesterol as one possible activation mechanism would be a fair representation of the data.

Reviewer #1 (Remarks to the Author):

The manuscript by Lo et al. focuses on the mechanistic activities Cnpy4, a novel regulator of the Hh pathway. In addition to using a murine transgenic line to demonstrate characteristic Hh related phenotypes including polydactyly, the authors identified Cnyp4 functions at the level of Smo to elevate sterol lipid composition of the ciliary membrane. As several recent studies have addressed the role of membrane lipid composition in terms of pathway modulation, this work would be of great interest to the Hedgehog community. However, there seem to be a few missed opportunities, that if addressed, could complete the story and increase the impact and biomedical relevance of the study.

1. In general, the Introduction does not set up the rationale for the study very well. There is background on the Hh transduction mechanism, but not on why examining this family member is important. Considering the link between cholesterol, Hh, and therapeutics that are widely available to reduce cholesterol levels, a more biomedically relevant introduction would increase the significance of the work.

We agree with Reviewer #1 that discussing the link between cholesterol, hedgehog (HH) signaling, and therapeutics that reduce cholesterol levels would improve the manuscript. We have significantly revised the introduction and discussion to include this information.

2. Where is the Cnpy4 transcript expressed throughout the embryo (temporally/spatially)? The endogenous expression pattern of Cnpy4 (or other family members) is not shown. Perhaps data from extended data figure 8, should also be added into main figures to partially address these questions. These data are important to support the conclusions made by authors.

As suggested, we have moved the immunofluorescence (IF) panels, which show the localization of overexpressed Flag-tagged human Canopy (CNPY) 4, from Supplementary Fig. 8 into Fig. 5.

With regards to *Cnpy4* transcript, we found it to be expressed in the mouse limb buds and have added this information to the manuscript text. In addition to the expression in the limb bud, we found that *Cnpy4* is also expressed in the long bones of the limbs (Rebuttal Fig. 1; also revised manuscript Supplementary Fig. 2). It has been previously reported that *Cnpy4* is widely expressed in mice, with the highest levels detected in the lung, thymus, uterus, and spleen[1], and we have added this information to the text.

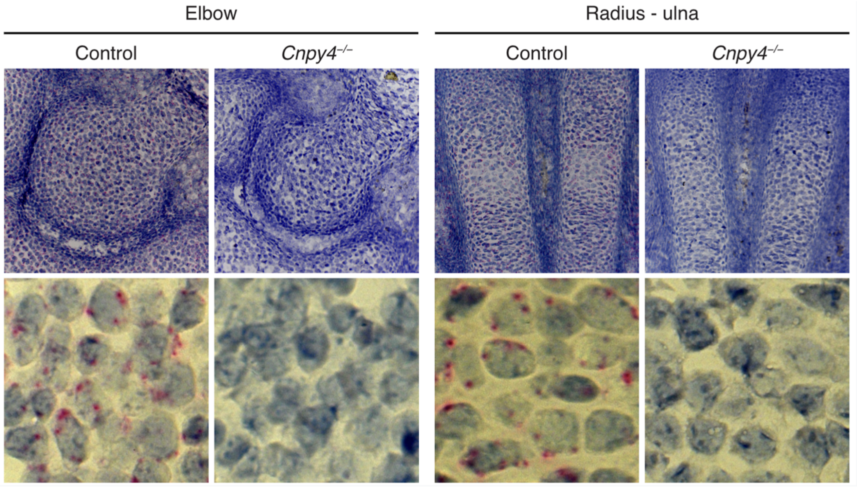

Rebuttal Fig. 1 *Cnpy4* transcript expression in the long bones of the limbs. Expression pattern of *Cnpy4* transcripts in the elbow (left panels) and the radius - ulna (right panels) at E14.5.

The question regarding the other CNPY family members and their possible interactions with CNPY4 is of interest, and we plan to pursue it in the future. We have already acquired mutant mice for *Cnpy1* and *Cnpy2*. In our initial analysis, we found that *Cnpy1* is expressed in the cerebellar region of developing mice, an expression pattern analogous to that found in zebrafish[2] (Rebuttal Fig. 2a). *Cnpy2* expression, on the other hand, is more widespread (Rebuttal Fig. 2b).

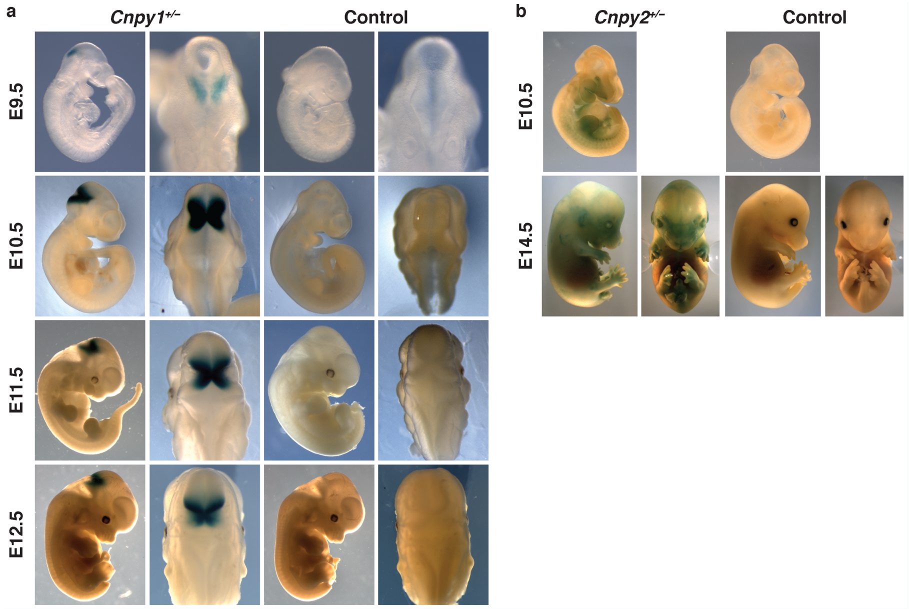

Rebuttal Fig. 2 Analysis of *Cnpy1* and *Cnpy2* expression using *lacZ* reporting lines. a, **b** *LacZ* staining of *Cnpy1* (**a**) and *Cnpy2* (**b**) heterozygous mutants compared to controls.

Our initial bulk RNA sequencing results indicate that the CNPY family members are either not expressed in the limb bud (*Cnpy1*) or have unaltered expression (*Cnpy2* and *Cnpy3*) in *Cnpy4*

knockout limb buds (Rebuttal Table 1). We plan to continue this analysis in the future as part of a separate study by performing additional bulk RNA sequencing.

Rebuttal Table 1: *Cnpy* transcript expression levels in *Cnpy4* knockout limb buds. Relative expression levels of *Cnpy* genes in *Cnpy4* knockout (dark grey rows) and control (light grey rows) limb buds with the calculated false discovery rate (FDR) (second row).

**Gene**	*Cnpy1*	*Cnpy2*	*Cnpy3*	*Cnpy4*
**KO vs. WT – FDR**	1	0.95	1	0
F-KO1010	2.8	7482.2	1892.3	6.5
F-KO3007	3.4	9328.2	1537.2	1.4
F-KO4004	2.8	9549.1	1952.2	1.8
F-KO4006	0	9142.6	1943.8	5.2
M-KO2003	0	7773.9	1813.8	2.3
M-KO2005	0	7391.6	1727.7	7.3
M-KO3004	0	8560.6	1666.7	6.7
M-KO4007	0.8	100002.7	2065.4	6.6
F-WT1006	0.8	7489.6	1874.9	1894.1
F-WT2008	0	8860.8	1606.9	2089.9
F-WT3002	0	5700.7	1879.7	2034.5
M-WT1001	0.7	8042.8	2074	1726
M-WT2002	0	6352.1	1850.5	1852.6
M-WT2004	4.6	7511.1	1970.2	1839.4
M-WT4005	0	9742.6	1646.4	1956.2

3. Previous reports have linked Cnpy genes to Fgf signaling. Is Fgf signaling maintained in Cnpy4-/- mutants? This seems highly relevant considering the interactions between FGF and SHH, and Gli3/Fgf during limb development.

Indeed, previous papers have linked the CNPY genes to fibroblast growth factor (FGF) signaling. This connection has been most thoroughly explored for CNPY1[2,3], less so for CNPY2[4], and not at all for CNPY3-5. Specifically, *cnpy1* was reported to positively regulate *fgf8*[2,3] in the midbrain-hindbrain region of zebrafish[2]. CNPY2 has a diverse set of roles, including, but not limited to, regulation of neurite outgrowth in mouse neuroblastoma cells and enhancement of angiogenesis and initiation of the PERK-CHOP pathway in human and mouse models[4–9]. With regard to FGF signaling, expression of CNPY2 in both mouse and human cells was shown to be positively correlated with stimulation with exogenous FGF21, although no mechanistic link was investigated[4]. Other CNPY family members have been reported to have diverse roles unrelated to FGF signaling. For example, CNPY3 was reported to alter the membrane levels of Toll-like

receptors in human cells[10–14], and CNPY5 may have a role in the folding of secretary proteins in the ER[15]. We have added this additional background information into the introduction of the revised version of the manuscript, as recommended by both Reviewers #1 and #2.

We probed for a possible connection between CNPY4 and FGF signaling by examining the effect of *Cnpy4* deletion on levels of FGF pathway genes via qRT-PCR (Rebuttal Fig. 3a, b) and *in situ* hybridization and *lacZ* expression (Rebuttal Fig. 3c). We did note a modest decrease in the levels of *Fgf8* expression in *Cnpy4*$^{-/-}$ samples via qRT-PCR (Rebuttal Fig. 3a), and we plan to explore the effect of CNPY4 on this signaling pathway in future studies. Our *in situ* hybridization analysis showed that there were *Cnpy4*$^{-/-}$ samples with both slight increases and expanded expression of the FGF target *Etv5* (but no major changes to *Fgf8*), as well as others with decreased *Fgf8* and *Etv5* (Rebuttal Fig. 3c).

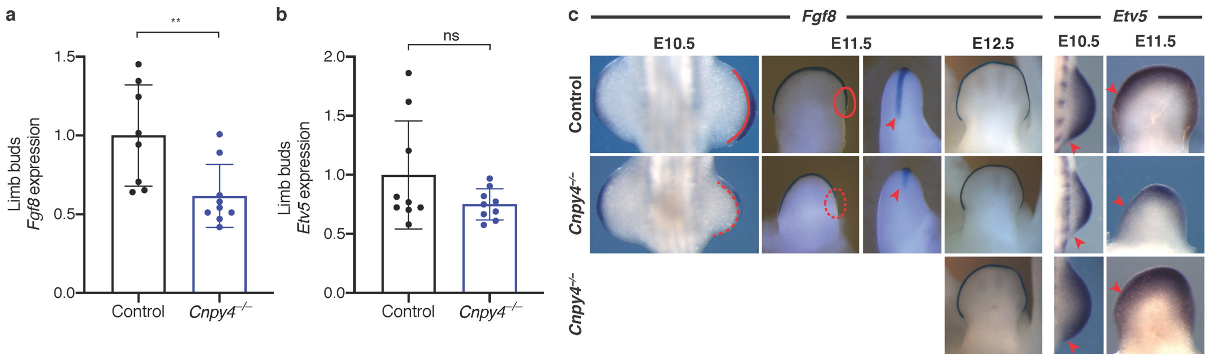

Rebuttal Fig. 3 *Fgf8* and *Etv5* transcript expression in *Cnpy4* mutants. a, **b** Expression levels of *Fgf8* (**a**) and *Etv5* (**b**) mRNA transcripts in control and *Cnpy4* mutant limb buds as measured by qRT-PCR. Data represent the mean ± SD (n = 9 embryos). Significance was calculated using a Mann-Whitney analysis with ns $p_{Etv5} = 0.3865$, $^{**}p_{Fgf8} < 0.005$. **c** Expression levels of *Fgf8* and *Etv5* mRNA transcripts in control and *Cnpy4* mutant limb buds by whole mount *in situ* hybridization and *lacZ* expression (using *Cnpy4;FGF8*^{lacZ} line at E11.5 and E12.5). Limb buds displayed a variation of expression patterns (lines, circles, and arrows) with some having reduced expression of *Fgf8* and *Etv5* (middle row) and others an expanded expression of *Etv5* but no differences in *Fgf8* expression (bottom row).

We additionally examined the effect of *Cnpy4* knockdown on downstream FGF signaling by measuring the levels of FGF-dependent phosphorylation of Akt and ERK in control and *Cnpy4*$^{-/-}$ MEF cells. We found that maximum magnitude of Akt and ERK phosphorylation in *Cnpy4*$^{-/-}$ MEFs (t = 10 min) was statistically not different than that measured in the control MEFs, although the signal decayed more quickly (t = 60, 90, and 120 min). However, these effects on the decay were modest compared to the CNPY4-dependent modulation of HH signaling; therefore, we concluded that the phenotypes observed in *Cnpy4* mutants are predominantly due to altered HH signaling. These data have now been added to the manuscript (Rebuttal Fig. 4; also revised manuscript Supplementary Fig. 6).

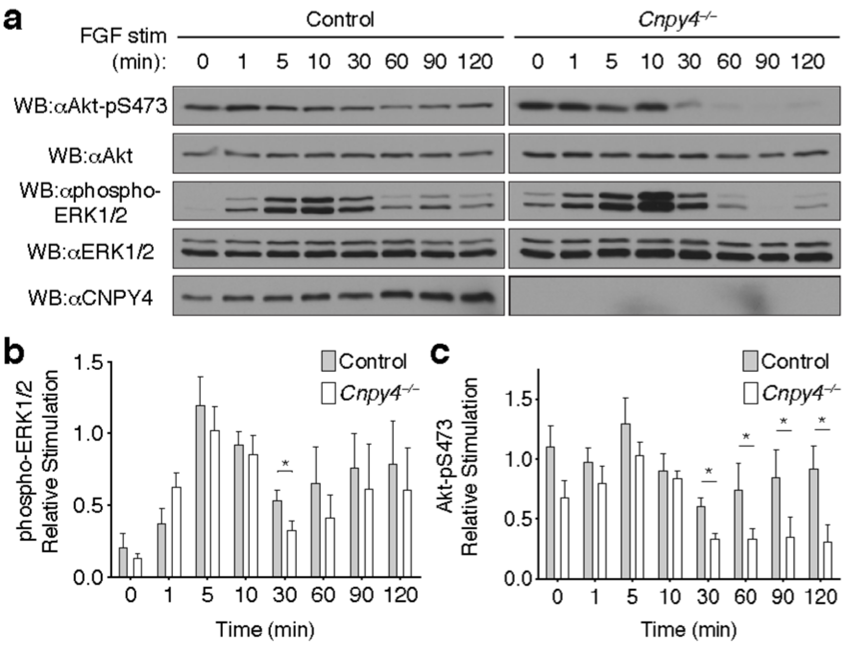

Rebuttal Fig. 4 Absence of *Cnpy4* diminishes FGF signaling. a FGF1 stimulation of control and *Cnpy4*$^{-/-}$ MEF cells. Protein levels in lysates were normalized using a BCA assay and were detected using the indicated antibodies by Western blot analysis. **b**, **c** Quantifications of Akt-pS473 (**b**) and phospho-ERK (**c**) upon FGF1 stimulation of MEF cells. Data were doubly normalized against the corresponding non-phosphorylated species as a loading control and to the zero time-point. Data represent the mean ± SEM (n = 7 from seven independent experiments). Significance was calculated using a Welch's t-test with *$p < 0.05$.

4. Was genetic background of mice considered in regard to the variable phenotype of Cnpy4-/- mice? There is precedence for variable expression of ciliary genes, and this can be observed on different backgrounds. Furthermore, this could offer an alternative hypothesis of genetic modifiers impacting expression and eventual phenotype.

We appreciate this suggestion and did indeed take into consideration the genetic background of the mice. The *Cnpy4* heterozygous knockout chimera were produced such that the resulting offspring were *129-C57BL/6J* hybrids. We backcrossed the offspring into four different strains of mice: *C57BL/6J* ,129/SvJ, FVB/NJ and ICR (CD-1). Starting from the 3rd generation, we did not observe any mutants in the FVB/NJ or in the ICR (CD-1) backcrosses after embryonic day 8 (P8) (Rebuttal Table 2), indicating that in these backgrounds, *Cnpy4* is essential for early development. We continued the backcrosses of these lines and cryopreserved them at the 10th generation to enable us to pursue future studies of the role of *Cnpy4* in early development. *Cnpy4* mutants were seen at the expected Mendelian ratio in *C57BL/6J* and 129/SvJ backgrounds, and the variable phenotype of the *Cnpy4* mutants persisted in these strains, with both poly- and oligodactyly. We decided to use *C57BL/6J* for the current study, as the mice in this line breed better than the 129, and we continued the backcrosses with this line. At the 10th generation of backcrossing, the *Cnpy4*$^{-/-}$ phenotype was still variable (Rebuttal Table 2). Therefore, it is clear that the variable

phenotype we report is not a result of a mixed genetic background. The information regarding the background of the mice has now been added to the methods section of the revised manuscript.

Rebuttal Table 2: *Cnpy* mutants in tested mice strains. Non-Mendelian ratios of embryos were observed from heterozygous *Cnpy4* mice intercross on FVB and CD1 genetic backgrounds (upper rows), while the expected Mendelian ratios were observed from heterozygous *Cnpy4* mice on 129 and *C57/B6* genetic background (lower rows).

Strain	Examined litters (n)	Examined embryos (n)	*Cnpy4*$^{-/-}$	*Control*	*Het*	KO Phenotypes
FVB	14	117	6	38	73	6/6 resorbed
		Percentage	5.1	32.5	62.4	
CD1	5	39	2	9	28	2/2 resorbed
		Percentage	5.1	23.1	71.8	
129	14	111	19	29	63	Variable
		Percentage	17.1	26.1	56.8	
C57/B6	61	381	89	90	202	Variable
		Percentage	23.5	23.5	53	

5. Onset of the polydactylous phenotypes, especially those in ciliary mutants, has been linked to a loss of the Gli3 repressor. Is the Gli3R isoform lost in Cnpy4-/- mutants?

Indeed, polydactyly has previously been linked to a loss of the *Gli3* repressor[16]. In response to this question, we assessed the effect of *Cnpy4* knockdown on *Gli3* transcript expression by qRT-PCR (Rebuttal Fig. 5a) and whole mount *in situ* hybridization (Rebuttal Fig. 5b). We found *Gli3* expression to be unchanged via qRT-PCR but variable at E10.5 via *in situ* hybridization. This might reflect the polydactyly/oligodactyly phenotypic spectrum that we observe. We have chosen to not include these data in the current paper, which focuses on mechanisms by which CNPY4 affects signaling, but we are planning to conduct a full developmental characterization in a subsequent study.

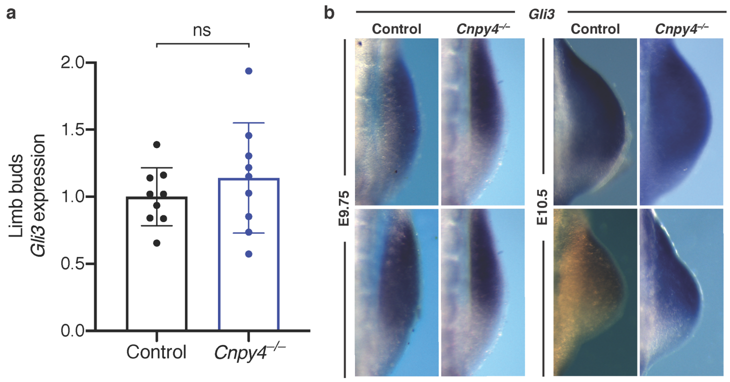

Rebuttal Fig. 5 *Shh* and *Gli3* transcript expression in *Cnpy4* mutants. a Expression levels of *Gli3* mRNA transcripts in control and *Cnpy4* mutant limb buds as measured by qRT-PCR. Data represent the mean ± SD (*n* = 9 embryos). Significance was calculated using a Mann-Whitney

analysis with ns p_{Gli3} = 0.3739. **b** Expression levels of *Gli3* mRNA transcripts in control and *Cnpy4* mutant limb buds as evaluated by *in situ* hybridization.

6. While it is understandable why the mechanistic studies were carried out under in vitro conditions, there is no attempt to test the suggested mechanism in vivo. For example, can the hyperactivity of the Hh pathway and subsequent Hh-related phenotypes in Cnpy4-/- mice be mitigated by reducing cholesterol production genetically or pharmacologically?

We appreciate these suggestions and intend to pursue them in our next series of experiments to be published in a subsequent paper. The events of the past year, including the pandemic and a pinworm infection in our colony, have precluded us from doing these experiments for the current manuscript, but they will fit well into the fuller *in vivo* analysis that we have planned.

Reviewer #2 (Remarks to the Author):

Lo et al. report CNPY4 functions in the regulation of the Hh pathway. They demonstrate that Cnpy4 KO mice showed polydactyly, which is typically observed in Hh-misregulated animals. In KO mice and siRNA-mediated knockdown cells, Hh hyperactivation is also confirmed. Although the downregulation of Cnpy4 does not affect general ciliary morphology and trafficking, they found that cellular sterol level is abnormally upregulated in Cnpy4-knockdown condition. In general, the manuscript is written and presented well, and features logical experimental designs (except Fig. 4).

The main criticism would be that, while the authors found a striking Hh-misregulated phenotype in knockout mice, the exact molecular mechanism of Cnpy4 for Hh signaling is still poorly characterized. Accordingly, this reviewer thinks that Cnpy4 is simply one of the dozens of genes involved in the HH pathway. It also does not make a great deal of sense that Cnpy4 as a lipid binding protein plays a role on lipids without direct interaction. In short, the effect of Cnpy4 KO/KD on sterol regulation should be investigated more deeply. For example, whether Cnpy4 downregulates general cholesterol level and/or targeting of cholesterol into specific organelles should be clarified. In addition, the following experiments are suggested to be performed to support the current dataset shown in Fig. 4 a-c.

1. Quantification of PFO at primary cilia in WT and KD cells

Per the Reviewer's suggestion, we have utilized an NIH3T3 cell line stably expressing ARL13B-GFP, which stains the primary cilia, in order to quantify PFO* fluorescence intensity at the cilia of WT and KD cells. We found that PFO* intensity increased both at the cilium and rest of the cell equally in KD cells (Rebuttal Fig. 6; also revised manuscript Fig. 5b, c).

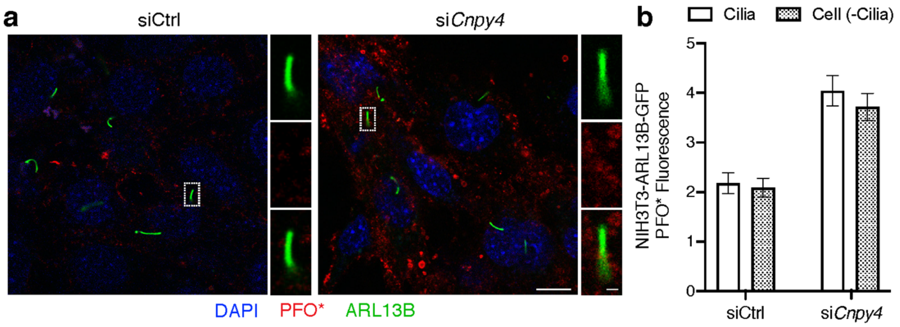

Rebuttal Fig. 6 CNPY4 modulates levels of accessible cholesterol. a Immunofluorescence-based staining of primary cilia (ARL13B-GFP, green), accessible cholesterol (PFO*-AF647, red), and nuclei (DAPI, blue) in ciliated NIH3T3 cells stably expressing ARL13B-GFP, treated with *Cnpy4* or control siRNA. The scale bar represents 10 μm. Cilia scale bar represents 1 μm. **b** Quantification of PFO* fluorescence intensity at the cilia and in the rest of the cell done measured

in FIJI. Error bars represent standard error of the mean (n = 104 siCtrl cells and n = 116 si*Cnpy4* cells from three independent experiments).

2. PFO staining in WT and KO embryos

Currently, use of the PFO* probe is limited to staining of live cells[17,18]; as the embryos are fixed, this technical limitation prevents us from staining control and KO embryos using this probe. As the technology develops further, we will work on staining embryos for future studies.

3. PFO staining with Cnpy4 overexpression in WT and KD cells

We appreciate this suggestion but believe that the other points, specifically point 4, raised by this Reviewer enable us to better connect CNPY4 and cholesterol and address the concern over the specificity of CNPY4's effect on HH signaling through modulation of cholesterol. We have therefore focused our efforts on addressing this point.

4. Depletion of cholesterol (for example by beta-cyclodextrin) in KD cells to see if HH activity goes back to normal level (functional rescue to confirm the functional specificity of Cnpy4)

Following this recommendation, we have examined the effect of *Cnpy4* knockdown on HH signaling when cholesterol is depleted. We utilized a combination of beta-cyclodextrin as well as lovastatin[19] to deplete membrane cholesterol before knocking down *Cnpy4* and stimulating cells with either SAG or SHH. Strikingly (and as predicted by the Reviewer), we found that in the absence of cholesterol, *Cnpy4* knockdown no longer had a potentiating effect on HH signaling (Rebuttal Fig. 7; also revised manuscript Fig. 4d, e). These results underscore our findings of the connection between the levels of membrane cholesterol and the CNPY4-dependent modulation of the HH pathway.

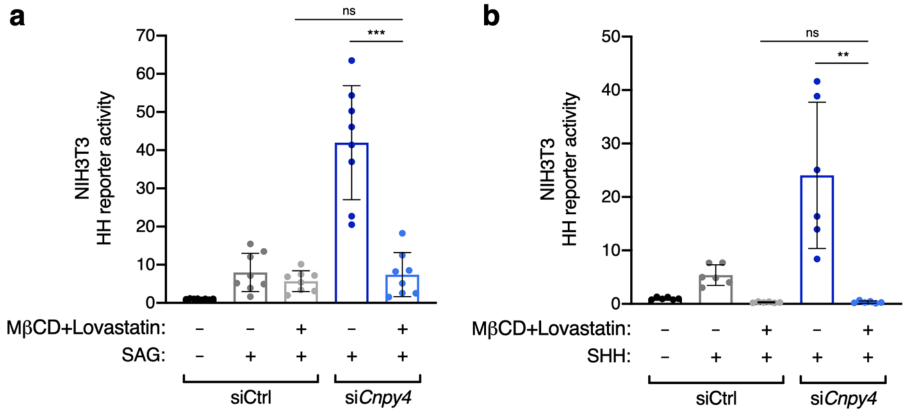

Rebuttal Fig. 7 CNPY4 knockdown does not potentiate HH signaling under conditions of membrane cholesterol depletion. a, b Luciferase reporter assay in ciliated NIH3T3 cells, incubated with MCβD and lovastatin prior to control or *Cnpy4* siRNA treatment and SAG (**a**) or SHH (**b**) stimulation. Data were normalized to the average value of control siRNA treated cells

stimulated with vehicle. Data represent the mean ± SD (n = 9 from three biological and three technical replicates). Significance was calculated using a Mann-Whitney analysis **p <0.005, ***p > 0.001.

Other miscellaneous points:

1. Introduction should clarify what is already known about Cnpy4.

We have expanded our introduction to review what is known about both CNPY4 and the other members of the CNPY family.

2. The molecular mechanism of Cnpy4-mediated Hh signaling should be examined in more detail. It seems that Cnpy4 inhibition does not affect Smo entry upon Hh ligands. The level of Smo activation and Sufu inactivation should be compared in WT and KD cells.

Reviewer #2 raises some excellent points here on how we could examine the molecular mechanism of CNPY4-mediated HH signaling in more depth. However, at this point, there is no agreed upon assay or method by which we could examine SUFU inactivation, as the output of SUFU activation (or inactivation) remains unclear. With regards to SMO, it is our understanding that, unlike other GPCRs, there are no widely used direct activity assays for SMO that truly recapitulate HH signaling biology. Therefore, there are currently no straightforward experimental approaches by which we can assess the differences in SMO activation in WT and KD cells. However, we plan to examine how CNPY4 affects the activation state of HH pathway genes through genetic crosses in future studies and have already acquired mutant mice for several of these genes.

3. Line 206: possible typo “it possible that”

We have edited the manuscript to correct this error.

Reviewer #3 (Remarks to the Author):

This study by Lo et al identifies Canopy 4 (CNPY4) as a regulator of the Hedgehog signaling pathway. This is an interesting and unexpected finding that should be published, however there are some issues. As described in the Abstract, the paper has two aspects, one of which is characterizing CNPY4 knockout animals in terms of Hedgehog pathway signaling, and the other of which is a speculative proposal for how CNPY4 may be acting through modulating membrane cholesterol levels. The first aspect is well-developed and the data are convincing, I need no further convincing that CNPY4 is important for Hedgehog signaling through SMO and for proper digit development! However, the second aspect is too speculative and should either be toned down or more work needs to be done.

We appreciate the Reviewer's interest in our work. We believe that with additional data, we have strengthened the second part of our study that discusses the role of cholesterol in CPNY4-mediated effects, as detailed below.

There are several points of concern:

1) The Abstract states (and it is repeated throughout the paper) that knockdown of CNPY4 "elevates membrane levels of accessible… cholesterol". All of this is based on one set of sub-figures in Fig. 4a-c using a probe for plasma membrane accessible cholesterol. Much more would need to be done to refine this correlation. Does overall cholesterol increase or is it just the pool of accessible cholesterol?

As discussed above in response to Reviewer #2, we have expanded our investigation of this mechanism to strengthen our conclusions and examined whether *Cnpy4* knockdown influences HH signaling when cholesterol is depleted. As shown in Rebuttal Fig. 7 (also revised manuscript Fig. 4d, e), depletion of membrane cholesterol by treatment of cells with a combination of beta-cyclodextrin and lovastatin eliminates the potentiating effect of *Cnpy4* knockdown on HH signaling. These results are consistent with our findings of the connection between the levels of membrane cholesterol and the CNPY4-dependent modulation of the HH pathway.

To further address this Reviewer's concern, we have investigated the effect of CNPY4 on accessible cholesterol in more depth. We quantified the levels of accessible cholesterol, as measured by the PFO* probe, in the whole cell and cilia (please see Rebuttal Fig. 6 above; also revised manuscript Fig. 5b, c), and we measured the levels of cholesterol and select cholesterol precursors in control and *Cnpy4* knockdown (KD) NIH3T3 cells via a mass-spectrometry based sterolomics approach (Rebuttal Fig. 8; also revised manuscript Supplementary Fig. 11). Through these evaluations, we determined that overall accessible cholesterol levels are specifically raised in KD cells (Rebuttal Fig. 6), with only modest changes in total cholesterol levels (Rebuttal Fig.

8f). Furthermore, we observed that the levels of most of the cholesterol precursor sterols included in our analysis, in particular those known to stimulate the HH pathway, are unchanged in KD cells (Rebuttal Fig. 8d) – again pointing to a specific effect of *Cnpy4* knockdown on the levels of free membrane cholesterol. Notably, there were two sterol species that were statistically significantly altered in KD cells, 7-dehydrodesmosterol (7-DHD) and 7-dehydrocholesterol (7-DHC) (Rebuttal Fig. 8c, e). While these changes could indicate changes to the upstream cholesterol biosynthesis enzyme DHCR7[20], which was recently shown to be positively correlated with HH activation, the changes observed overall do not point to CNPY4 having a central role in cholesterol biosynthesis.

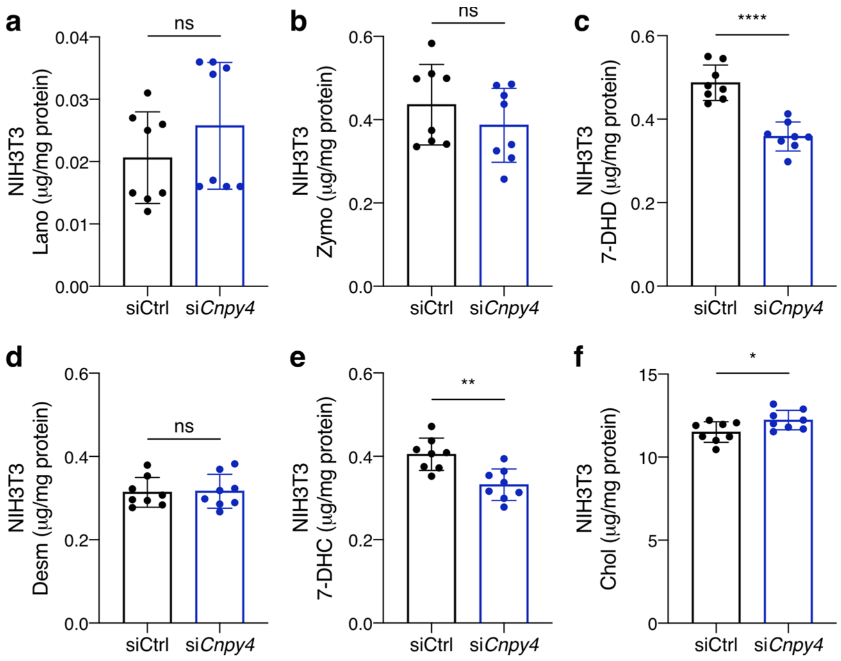

Rebuttal Fig. 8 Effect of CNPY4 knockdown on the levels of sterol precursors in cholesterol biosynthesis. a–f Mass spectrometry-based sterolomics analysis of the cell lysates from NIH3T3 cells treated with *Cnpy4* or control siRNA, showing levels of cholesterol precursors: lanosterol (**a**), zymosterol (**b**), 7-dehydrodemosterol (**c**), desmosterol (**d**), 7-dehydrocholesterol (**e**), and cholesterol (**f**). Data represent the mean ± SD ($n = 8$). Significance was calculated using a Welch's t-test with ns $p > 0.05$, *$p < 0.05$, **$p < 0.005$, ****$p < 0.0001$.

2) In Figure 4D, the authors draw CNPY4 in the ER and imply that it modulates cholesterol synthesis. There is no data for this. Does CNPY4 affect the activation of SREBP transcription factors and through that affect cholesterol synthesis? Or does it activate some transport mechanism between the ER and plasma membrane?

As shown above, our mass-spectrometry based sterolomics suggest that CNPY4 is not a master regulator of lipid biosynthesis. We have revised our schematic from Fig. 4D (now Fig. 5D) to remove this implication. We have yet to investigate the possibility that CNPY4 affects SREBP

transcription or that it activates a transport mechanism between the ER and plasma membrane; we plan to investigate this question in future studies.

I think keeping the focus of the paper on the remarkable effect of CNPY4 knockdown on digit development and merely pointing out a correlation with accessible cholesterol as one possible activation mechanism would be a fair representation of the data.

Based on this suggestion, we have revised our manuscript to tone down our speculations on the relationship between CNPY4 and the accessibility of membrane cholesterol. This topic is now an active area of research in our laboratories as we work to understand this mechanism in more depth.

References

1. Konno, K. *et al.* A molecule that is associated with Toll-like receptor 4 and regulates its cell surface expression. *Biochem. Biophys. Res. Commun.* **339**, 1076–1082 (2006).

2. Hirate, Y. & Okamoto, H. Canopy1, a Novel Regulator of FGF Signaling around the Midbrain-Hindbrain Boundary in Zebrafish. *Curr. Biol.* **16**, 421–427 (2006).

3. Matsui, T. *et al.* Canopy1, a positive feedback regulator of FGF signaling, controls progenitor cell clustering during Kupffer's vesicle organogenesis. *Proc. Natl. Acad. Sci.* **108**, 9881–9886 (2011).

4. Do, H. T. *et al.* Fibroblast Growth Factor-21 (FGF21) Regulates Low-density Lipoprotein Receptor (LDLR) Levels in Cells via the E3-ubiquitin Ligase Mylip/Idol and the Canopy2 (Cnpy2)/Mylip-interacting Saposin-like Protein (Msap). *J. Biol. Chem.* **287**, 12602–12611 (2012).

5. Guo, J. *et al.* A secreted protein (Canopy 2, CNPY2) enhances angiogenesis and promotes smooth muscle cell migration and proliferation. *Cardiovasc. Res.* **105**, 383–393 (2015).

6. Dou, Y. *et al.* The CNPY2 enhances epithelial-mesenchymal transition via activating the AKT/GSK3β pathway in non-small cell lung cancer. *Cell Biol. Int.* **42**, 959–964 (2018).

7. Hong, F. *et al.* CNPY2 is a key initiator of the PERK-CHOP pathway of the unfolded protein response. *Nat. Struct. Mol. Biol.* **24**, 834– 839 (2017).

8. Ito, S. *et al.* CNPY2 inhibits MYLIP-mediated AR protein degradation in prostate cancer cells. *Oncotarget* **9**, 17645–17655 (2018).

9. Bornhauser, B. C., Olsson, P.-A. & Lindholm, D. MSAP Is a Novel MIR-interacting Protein That Enhances Neurite Outgrowth and Increases Myosin Regulatory Light Chain. *J. Biol. Chem.* **278**, 35412–35420 (2003).

10. Takahashi, K. *et al.* A protein associated with Toll-like receptor (TLR) 4 (PRAT4A) is required for TLR-dependent immune responses. *J. Exp. Med.* **204**, 2963–2976 (2007).

11. Wakabayashi, Y. *et al.* A protein associated with toll-like receptor 4 (PRAT4A) regulates cell surface expression of TLR4. *J. Immunol.* **177**, 1772–1779 (2006).

12. Kiyokawa, T. *et al.* A single base mutation in the PRAT4A gene reveals differential interaction of PRAT4A with Toll-like receptors. *Int. Immunol.* **20**, 1407–1415 (2008).

13. Hart, B. E. & Tapping, R. I. Cell Surface Trafficking of TLR1 Is Differentially Regulated by the Chaperones PRAT4A and PRAT4B. *J. Biol. Chem.* **287**, 16550–16562 (2012).

14. Shibata, T. *et al.* PRAT4A-dependent expression of cell surface TLR5 on neutrophils, classical monocytes and dendritic cells. *Int. Immunol.* **24**, 613–623 (2012).

15. Schildknegt, D. *et al.* Characterization of CNPY5 and its family members. *Protein Sci.* **28**, 1276–1289 (2019).

16. Zuniga, A. & Zeller, R. Gli3 (Xt) and formin (ld) participate in the positioning of the polarising region and control of posterior limb-bud identity. *Development* **126**, 13–21 (1999).

17. Das, A., Brown, M. S., Anderson, D. D., Goldstein, J. L. & Radhakrishnan, A. Three pools of plasma membrane cholesterol and their relation to cholesterol homeostasis. *eLIFE* **3**, 19316 (2014).

18. Kinnebrew, M. *et al.* Cholesterol accessibility at the ciliary membrane controls hedgehog signaling. *eLIFE* **8**, e50051 (2019).

19. Myers, B. R. *et al.* Hedgehog Pathway Modulation by Multiple Lipid Binding Sites on the Smoothened Effector of Signal Response. *Dev. Cell* **26**, 346–357 (2013).

20. Findakly, S. *et al.* Sterol and oxysterol synthases near the ciliary base activate the Hedgehog pathway. *J. Biol. Chem.* **220**, 1019 (2020).

REVIEWERS' COMMENTS

Reviewer #1 (Remarks to the Author):

The authors have sufficiently addressed this Reviewer's concerns.

Reviewer #2 (Remarks to the Author):

The authors addressed my original concerns properly and professionally.

Reviewer #3 (Remarks to the Author):

I thank the authors for their additional efforts to address my concerns that their claims that CNPY4 acted through the modulation of cholesterol levels was premature. The new experiments showing that depletion of cholesterol by cyclodextrin treatment eliminates the potentiating effect of CNPY4 knockdown on HH signaling, as well as measuring an increase in accessible cholesterol levels in the cilia of CNPY4 knockdown cells, strengthen their proposed cholesterol connection. Of course, these results raise more questions, and I look forward to their future work addressing the connection between CNPY4 and membrane accessible cholesterol levels.
